# Bayesian Analysis of ANOVA and Mixed Models on the Log-Transformed Response Variable

**DOI:** 10.1007/s11336-021-09769-y

**Published:** 2021-06-04

**Authors:** Aldo Gardini, Carlo Trivisano, Enrico Fabrizi

**Affiliations:** 1 Dipartimento di Scienze Statistiche ‘P. Fortunati’, Università di Bologna, Bologna, Italy; 2 Università Cattolica del S. Cuore, Milano, Italy

**Keywords:** Generalized inverse Gaussian, Markov chain Monte Carlo, Log-normal distribution, Response times

## Abstract

The analysis of variance, and mixed models in general, are popular tools for analyzing experimental data in psychology. Bayesian inference for these models is gaining popularity as it allows to easily handle complex experimental designs and data dependence structures. When working on the log of the response variable, the use of standard priors for the variance parameters can create inferential problems and namely the non-existence of posterior moments of parameters and predictive distributions in the original scale of the data. The use of the generalized inverse Gaussian distributions with a careful choice of the hyper-parameters is proposed as a general purpose option for priors on variance parameters. Theoretical and simulations results motivate the proposal. A software package that implements the analysis is also discussed. As the log-transformation of the response variable is often applied when modelling response times, an empirical data analysis in this field is reported.

**Supplementary Information:**

The online version contains supplementary material available at 10.1007/s11336-021-09769-y.

## Introduction

The analysis of variance (ANOVA) is a popular tool for analyzing experimental data in psychology as in many other research fields. The assumptions underpinning the standard ANOVA are rather restrictive as response variables may not be normally distributed (Micceri, 1989; Blanca et al., 2017), sample sizes can be rather small (Button et al., 2013), and the assumption of independence between observations may fail when data follow a multi-level structure (Gelman and Hill, 2007). The latter problem is often involved in the analysis of data from within subjects or mixed (within and between subjects) experimental designs, whose popularity is increasing (Charness et al., 2012; Wedel and Dong, 2020).

For these reasons, ANOVA analyses are often conducted in the more general framework of mixed models, either linear, nonlinear or linear but specified on a transformation of the response variable (Boisgontier and Cheval, 2016; Singmann and Kellen, 2019). In this paper, a special attention is devoted to linear mixed models specified on the $\log$ of the response variable, a popular solution to overcome non-normality which is often applied in psychology. A notable example in this direction is provided by the analysis of response times (RT), a positive variable that turns out to be skewed and with a variance that typically increases with the mean. Recent reviews on RT modelling can be found in Lee and Chen (2011) and De Boeck and Jeon (2019). The log-transformation of RT is considered in Thissen (1983); Van Breukelen (2005); van der Linden (2006); Loeys et al. (2011); Rouder et al. (2015) among many others. The interest in modelling RT is rising also in educational sciences (van der Linden 2009) where it received an impetus from the computerization of educational testing.

Of course, the log-transformation is not the only way to deal with data non normality, and it does not always go without problems (Feng et al., 2013; Changyong et al., 2014). Nonetheless, in this paper we assume that the transformed data are normally distributed and focus on specific inferential problems related to linear mixed models on log-transformed data.

The back-transformation of the results to the original data scale is one of the major issues faced by applied scientists when a model is estimated on transformed data. With reference to the analysis of RT, it is often needed to compare RT across individuals, groups or items on the their raw scale (Posner, 1978; Lo and Andrews, 2015).

The Bayesian approach to ANOVA offers several advantages with respect to standard frequentist methods, including a flexible, unified treatment of linear and nonlinear mixed models, the simpler interpretation of p-values and credible intervals, the possibility of making inference not only for model parameters but also for their transformations (Kruschke, 2013; Wagenmakers et al., 2018b). In particular, we can immediately carry out inference also for back-transformed quantities, such as conditional means.

The need to specify priors incorporating subjective information often hinders the recourse to Bayesian ANOVA by applied researchers (Rouder et al., 2012). For this reason, recently proposed software packages such as BANOVA and JASP implement default priors that can be overlooked by data analyzers that do not want to incorporate actual prior information (Dong and Wedel, 2017; Wagenmakers et al., 2018a). Unfortunately, inference relying on the default priors considered by these packages (and on most of those in the literature) for the variance components can run into problems, when mixed models specified on the log of the response variable are used. Specifically, if we let $y>0$ be the variable we target, $w=\log (y)$ and we focus on the estimation of $\mathbb {E}(y)$ or on the prediction of *y* values for a given set of observed covariates, it can easily be shown that posterior distributions, although formally well defined, have no finite moments and can thereby lead to wrong inferences as common posterior summaries such as posterior means and standard deviations are undefined. Inferences on expectations on the data actual scale are not equivalent to those conducted at the transformed scale. As a simple example, let us consider the case of the comparison of two groups mean response values. The equality of the means on the log scale does not implies the equality of the means on the raw scale as the latter are functions also of the scale parameters (see Changyong et al., 2014 for further discussion).

The main contribution of this paper is to propose the Generalized Inverse Gaussian (GIG) distribution as default prior for the variance components of linear mixed models. Endowed with suitably selected hyper-parameters, GIG priors lead to results virtually equal to those obtained adopting currently default choices when the problem of back-transforming quantities estimated on the log-scale is not involved and guarantee correct inferences when it is. The GIG is a flexible family of three parameters distributions with positive support that encompasses several well-known special cases (Gamma and Inverse Gamma, among others). More importantly, they allow for simple expressions of the conditions on prior parameters that guarantee the existence of posterior moments; eventually, their conjugacy with the normal allows for the implementation of fast Gibbs sampling algorithms to explore the posterior distributions of interest.

This work builds upon earlier contributions of Fabrizi and Trivisano (2012; 2016) but represents a significant addition to their results as deriving conditions for the existence of posterior moments goes along different lines and is definitely more challenging in the context of mixed models with respect to the fitting of a log-normal distribution and a linear regression model considered by these authors. The reason is that, when introducing random effects, relevant posterior distributions are not available in a closed form anymore.

The structure of the paper is as follows. Section 2 provides a theoretical background: we first introduce our notation, some known results about the Bayesian analysis of the linear mixed model and the GIG distribution. In Sect. 3, we introduce the main theoretical result, that is the required conditions on the GIG parameters that allow for the existence of posterior moments for functionals of the parameters such as $\mathbb {E}(y)$ or the predictive distribution; this Section contains also a discussion on the properties of these posterior distributions when associated with other classes of prior distributions for the variance components. In Sect. 4, we discuss how to set the parameters of the GIG priors uninvolved in the existence of posterior moments and the Gibbs sampling algorithms needed to explore posterior distributions. Section 5 reports some results from the simulation studies we performed. In Sect. 6, we illustrate a real data application taken from cognitive science literature. In Sect. 7, the obtained results, their scope and limitations are discussed, along with some possible directions for further research. Eventually, Sect. 8 offers some concluding remarks. More details on the simulation results and additional, complementary, technical results can be found in the on-line supplementary material.

## Notation and Preliminary Results

In this section, we first introduce a general specification for the linear mixed model on the log-scale along with a basic result conditional on the variance components. Then, we shortly describe the GIG distribution that will be considered in further analyses.

### The Log-Normal Mixed Model

Let us consider a *n*-dimensional vector of strictly positive responses $\mathbf {y}$; once defined $\mathbf {w}=\log \mathbf {y}$, a linear mixed model is assumed:

$$\begin{aligned} \mathbf {w}= \mathbf {X}\varvec{\beta }+\mathbf {Zu}+\varvec{\varepsilon }, \end{aligned}$$

where $\varvec{\beta }\in \mathbb {R}^p$ is a vector of fixed effects, $\mathbf {u}\in \mathbb {R}^m$ is a vector of random effects and $\varvec{\varepsilon }\in \mathbb {R}^{n}$ is the vector of residuals. The design matrices are $\mathbf {X}\in \mathbb {R}^{n\times p}$, that is assumed to be full rank, and $\mathbf {Z}\in \mathbb {R}^{n\times m}$. The following Bayesian hierarchical model will be studied:

1 $$\begin{aligned} \begin{aligned}&\mathbf {w}|\mathbf {u}, \varvec{\beta }, \sigma ^2\sim \mathcal {N}_n\left( \mathbf {X}\varvec{\beta }+\mathbf {Zu}, \mathbf {I}_n\sigma ^2 \right) ;\\&\quad \mathbf {u}|\tau ^2_1,...,\tau ^2_q\sim \mathcal {N}_m\left( \mathbf {0}, \mathbf {D}\right) ,\ \mathbf {D}=\oplus ^q_{s=1}\mathbf {I}_{m_s}\tau _s^2. \end{aligned} \end{aligned}$$

Note that $q\ge 1$ random factors are allowed, so that *q* different variances related to the random components $\varvec{\tau }^2=(\tau ^2_1,...,\tau ^2_q)$ are included in the model. Therefore, it is possible to split the vector of random effects in $\mathbf {u}=[\mathbf {u}_1^T,...,\mathbf {u}_s^T,...,\mathbf {u}_q^T]^T$, where $\mathbf {u}_s\in \mathbb {R}^{m_s}$ with $\sum _{s=1}^q m_s=m$. The design matrix of the random effects can be partitioned too: $\mathbf {Z}=[\mathbf {Z}_1\cdots \mathbf {Z}_s\cdots \mathbf {Z}_q]$. We note that the design matrix of the random effects is not necessarily non-singular. For an introduction to the use of these models in the behavioral sciences framework, see, e.g., Jackman (2009, Chapter 7).

The introduced model is fairly general. All standard one and multi-ways ANOVA models as well as mixed models suitable for the analysis of repeated measures with both nested and crossed effects (Baayen et al., 2008) can be obtained as special cases. ANCOVA models, accounting for the effect of possible covariates, are also encompassed by (1), including models that allow for possible nonlinear effects of these covariates whose shape cannot be anticipated: in fact, spline regression can be represented by means of mixed models (see Crainiceanu et al., 2005). Equation (1) covers situations in which the assumption of independence between random effects fails, provided no additional parameter is involved: more specifically, if known positive matrices replace $\mathbf {I}_{m_s}$, (1) can be reparameterized to allow for correlated random effects (Hobert and Casella, 1996). On the contrary, models involving additional parameters describing the correlation between random effects are beyond the scope of (1) and thereby of our analysis. Nonetheless, a discussion of models in which correlated random effects are specified within grouping factors can be found in Sect. 7.

We now restate a known result on the posterior distribution of $\varvec{\beta }$ in order to set notations and define quantities that will be used later on.

#### Proposition 1

Considering the model (1) with a flat improper prior on $\varvec{\beta }$ then:

2 $$\begin{aligned} \varvec{\beta }|\sigma ^2, \varvec{\tau }^2, \mathbf {w}\sim \mathcal {N}_p\left( \bar{\varvec{\beta }},\mathbf {V}_\beta \right) , \end{aligned}$$

where:

$$\begin{aligned} \begin{aligned} \mathbf {V}_\beta&=\left( \frac{\left( \mathbf {X}^T\mathbf {X}\right) }{\sigma ^2}+ \mathbf {X}^T\mathbf {M}\mathbf {X}\right) ^{-1} ,\quad \bar{\varvec{\beta }}=\mathbf {V}_\beta \left( \frac{\mathbf {X}^T\mathbf {X}}{\sigma ^2}\hat{\varvec{\beta }}+\mathbf {X}^T\mathbf {M}\mathbf {X}\tilde{\varvec{\beta }}\right) ,\\ \mathbf {M}&=\left( \frac{\mathbf {V}^{-1}_Z}{\sigma ^2}-\frac{\mathbf {P}_Z}{\sigma ^2} \right) ,\quad \hat{\varvec{\beta }}= \left( \mathbf {X}^T\mathbf {X}\right) ^{-1}\mathbf {X}^T\mathbf {y},\quad \tilde{\varvec{\beta }}= \left( \mathbf {X}^T\mathbf {M}\mathbf {X}\right) ^{-1}\mathbf {X}^T\mathbf {M}\mathbf {y},\\ \mathbf {P}_Z&=\mathbf {Z}\left( \mathbf {Z}^T\mathbf {Z}\right) ^{-}\mathbf {Z}^T,\quad \mathbf {V}_Z^{-1}=\mathbf {Z}\left( \mathbf {Z}^T\mathbf {Z}\right) ^{-} \left( \left( \mathbf {Z}^T\mathbf {Z}\right) ^{-}+\frac{\mathbf {D}}{\sigma ^2}\right) ^{-1}\left( \mathbf {Z}^T\mathbf {Z}\right) ^{-}\mathbf {Z}^T, \end{aligned} \end{aligned}$$

and $\left( \mathbf {Z}^T\mathbf {Z}\right) ^{-}$ is the Moore–Penrose inverse of $\mathbf {Z}^T\mathbf {Z}$.

As anticipated in the introduction, in this paper we focus on the estimation of the expectation of *y* and on predictive distributions. Let the vectors $\tilde{\mathbf {x}}$, $\tilde{\mathbf {z}}$ represent a point in the covariates space conditionally on which we can be interested in estimating the expectation of *y*. More specifically, let us first consider:

3 $$\begin{aligned} \mathbb {E}\left[ \tilde{y}|\varvec{\beta },\sigma ^2,\varvec{\tau }^2\right] =\theta _m(\tilde{\mathbf {x}})=\exp \left\{ \tilde{\mathbf {x}}^T\varvec{\beta }+\frac{1}{2}\left( \sigma ^2+\sum _{s=1}^q \tau _s^2\right) \right\} , \end{aligned}$$

where the random effects are integrated out. We use the notation $\tilde{y}$ instead of *y* to emphasize we are working conditionally on $\tilde{\mathbf {x}}$ and $\tilde{\mathbf {z}}$. The expectation of *y* conditional on the random effects is another quantity that can be relevant in prediction problems:

4 $$\begin{aligned} \mathbb {E}\left[ \tilde{y}|\mathbf {u},\varvec{\beta },\sigma ^2 \right] =\theta _c(\tilde{\mathbf {x}},\tilde{\mathbf {z}})=\exp \left\{ \tilde{\mathbf {x}}^T\varvec{\beta }+\tilde{\mathbf {z}}^T\mathbf {u}+\frac{\sigma ^2}{2} \right\} . \end{aligned}$$

Finally, the posterior predictive distribution $p(\tilde{y}|\mathbf {y})$ and its posterior moments are further quantities to investigate. Note that:

5 $$\begin{aligned} p\left( \tilde{y}|\mathbf {y}\right) \propto \int _{\varvec{\Theta }} p\left( \tilde{y}|\varvec{\theta }\right) p\left( \varvec{\theta }|\mathbf {y}\right) \mathrm {d}\varvec{\theta }, \end{aligned}$$

where $\varvec{\theta }=(\varvec{\beta }, \mathbf {u}, \sigma ^2, \varvec{\tau }^2)$ and $\Theta$ is the parameter space. In practice, the posterior expectation $\mathbb {E}\left[ \tilde{y}|\mathbf {y}\right]$ might be used to predict unobserved values like missing values or unsampled units.

### The Generalized Inverse Gaussian Distribution

In this paper, we assume a GIG prior for the variance components. In general, a random variable *V* is GIG distributed, i.e., $V \sim GIG (\lambda ,\delta ,\gamma )$, if its density can be written as follows:

6 $$\begin{aligned} p(v)=\left( \frac{\gamma }{\delta } \right) ^\lambda \frac{1}{2K_\lambda (\delta \gamma )}v^{\lambda -1}\exp \left\{ -\frac{1}{2}\left( \delta ^2 v^{-1}+\gamma ^2 v\right) \right\} \mathbf {1}_{\mathbb {R}^+}. \end{aligned}$$

If $\delta > 0$, the permissible values for the other parameters are $\gamma \ge 0$ when $\lambda <0$, and $\gamma > 0$ if $\lambda =0$. If $\delta \ge 0$, then $\gamma$ and $\lambda$ should be strictly positive. The first reason to consider the GIG is that many important distributions may be obtained as special cases. For $\lambda >0$ and $\gamma > 0$, the $Gamma (\lambda ,\gamma ^2/2)$ distribution emerges as the limit when $\delta \rightarrow 0$. An inverse-gamma is obtained when $\lambda < 0$, $\delta >0$ and $\gamma \rightarrow 0$; an inverse Gaussian distribution is obtained when $\lambda =-\frac{1}{2}$. A uniform distribution over the range (0, *A*) for $\sqrt{V}$ implies that $p(v) \propto v^{-1/2}\mathbf {1}_{(0,A)}$, which may approximated by the density of a $GIG(0.5,\delta ,(2A^2)^{-1})$ with $\delta \rightarrow 0$ and truncated at $A^2$. This special case is relevant to discuss the uniform prior on the standard deviation advocated by Gelman (2006). For more details on the GIG distribution see Bibby and Sørensen (2003).

## Theoretical Results

In this section, we study the existence of moments for the posterior distributions of $\theta _m(\tilde{\mathbf {x}})$ and $\theta _c(\tilde{\mathbf {x}},\tilde{\mathbf {z}})$, defined in (3) and (4), and for the posterior predictive distribution $p(\tilde{y}|\mathbf {y})$ (5). As anticipated in the introduction, we assume GIG distributions for the hyper-parameters:

7 $$\begin{aligned} \sigma ^2\sim & {} GIG\left( \lambda _\sigma ,\delta _\sigma ,\gamma _\sigma \right) , \end{aligned}$$

8 $$\begin{aligned} \tau _s^2\sim & {} GIG\left( \lambda _{\tau ,s},\delta _{\tau ,s},\gamma _{\tau ,s}\right) ,\ \forall s. \end{aligned}$$

Before stating the main result of this section, let us define $\mathbf {L}_s\in \mathbb {R}^{p\times p}$ as a matrix whose entries are all 0s with the exception of the first $l \times l$ square block $\mathbf {L}_{s;1,1}$ where $l=p-rank\{ \mathbf {X}^T\left( \mathbf {I}-\mathbf {P_Z} \right) \mathbf {X}\}$ is the rank deficiency of $\mathbf {X}^T\left( \mathbf {I}-\mathbf {P_Z} \right) \mathbf {X}$ and it coincides with the number of columns of $\mathbf {X}$ that are included in $\mathbf {Z}$ too. To simplify the statement of our result, it is useful to work with a modified design matrix $\mathbf {X}_o$ obtained by placing the columns included in both $\mathbf {X}$ and $\mathbf {Z}$ as the first *l* columns, without loss of generality. Consequently, we note that $\mathbf {L}_{s;1,1}$ coincides with the inverse of upper left $l \times l$ block on the diagonal of $\mathbf {X}_o^T\left( \mathbf {Z}(\mathbf {Z}^T\mathbf {Z})^{-}\mathbf {C}_s (\mathbf {Z}^T\mathbf {Z})^{-}\mathbf {Z}^T\right) \mathbf {X}_o$, where $\mathbf {C}_s$ is the null matrix with the exception of $\mathbf {I}_{m_s}$ as block on the diagonal in correspondence to the *s*-th variance component of the random effect. Eventually, $\tilde{\mathbf {x}}_{o}$ is the covariate pattern of the new observation ordered consistently with $\mathbf {X}_o$.

### Theorem 1

If the normal linear mixed model in the log scale (1) is considered with the priors (7), (8), then, in order to compute the *r*-th, with $r>0$, posterior moment of $\theta _c(\tilde{\mathbf {x}},\tilde{\mathbf {z}})$, $\theta _m(\tilde{\mathbf {x}})$ and of $p(\tilde{y}|\mathbf {y})$, the following constraints on the prior parameters must be observed:

(i) $$\mathbb {E}\left[ \theta _c^r(\tilde{\mathbf {x}},\tilde{\mathbf {z}})|\mathbf {w}\right] $$ exists if $$\gamma _{\sigma }^2>r+r^2\tilde{\mathbf {x}}^T\left( \mathbf {X}^T\mathbf {X}\right) ^{-1}\tilde{\mathbf {x}}$$;
(ii) $\mathbb {E}\left[ \theta _m^r(\tilde{\mathbf {x}})|\mathbf {w}\right]$ exists if $\gamma _{\sigma }^2>r+r^2\tilde{\mathbf {x}}^T\left( \mathbf {X}^T\mathbf {X}\right) ^{-1}\tilde{\mathbf {x}}$ and $\gamma ^2_{\tau ,s}>r+r^2\tilde{\mathbf {x}}_{o}^T\mathbf {L}_s\tilde{\mathbf {x}}_{o}$, $\forall s$;
(iii) $\mathbb {E}\left[ \tilde{y}^r|\mathbf {y}\right]$ exists if $\gamma _{\sigma }^2>r^2+r^2\tilde{\mathbf {x}}^T\left( \mathbf {X}^T\mathbf {X}\right) ^{-1}\tilde{\mathbf {x}}$.

### Proof

See appendix. $$\square $$

Few comments on Theorem 1 are in order. We first note that the conditions on the existence of posterior moments depend only on constraints on the tail parameter $\gamma$. Moreover, $\theta _m(\tilde{\mathbf {x}})$ requires a condition on the parameters of all variance components prior, while $\theta _c(\tilde{\mathbf {x}},\tilde{\mathbf {z}})$ and the posterior predictive distribution need only a condition on $p(\sigma ^2)$, to ensure the finiteness of the posterior moments.

Statement (*i*) parallels the result by Fabrizi and Trivisano (2016) for the log-normal linear model: the square of the moment order *r* is multiplied by the leverage associated with $\tilde{\mathbf {x}}$, i.e., $\tilde{\mathbf {x}}^T\left( \mathbf {X}^T\mathbf {X}\right) ^{-1}\tilde{\mathbf {x}}$. The same condition on $\gamma _{\sigma }$ appears also for the moments of $\theta _m(\tilde{\mathbf {x}})$.

As far as the posterior predictive distribution, it concerns, i.e., case (*iii*), the existence of its posterior moments is related only to the term $\sigma ^2$. It must be noted that, unlike case (*i*), the quantity $r^2$ enters the condition as a separate term, making the value on the right side of the constraint rapidly increasing with the moment order. The result is in line with the higher variability that characterizes the posterior predictive distribution with respect to the posteriors of $\theta _c(\tilde{\mathbf {x}},\tilde{\mathbf {z}})$ and $\theta _m(\tilde{\mathbf {x}})$.

From Theorem 1 and its proof, it is apparent that, generally speaking, a prior containing an exponential term in the form $\exp \{-c\omega ^2 \}$ must be given as prior for the generic variance component $\omega ^2$, where *c* is set in order to have finite moments up to a pre-specified order. This helps us to understand which special cases within the GIG family and which distributions outside this group can be considered. Popular choices for priors on the variance components such as Jeffrey’s priors, uniform (both on the variance and on the standard deviation), half-t (including half-Cauchy) do not contain the exponential term in question. Other priors such as the inverse gamma (that is a special case of the GIG distribution when $\gamma \rightarrow 0$) or the log-normal, even if they contain an exponential term, cannot be used as this term does not go to 0 when $\omega ^2\rightarrow +\infty$.

Other distributions, outside the GIG family, can be considered as prior for the variance components, as for instance the half-normal $HN(\zeta )$, mentioned as reasonable prior for the standard deviation by Gelman (2006), provided that a small hyper-parameter $\zeta$ is chosen. In view of Theorem 1, it can be shown that, for example, the prior $\sigma \sim HN(\zeta _\sigma )$ should be specified in compliance with the following constraint:

$$\begin{aligned} \zeta _\sigma <\sqrt{\frac{1}{r+r^2\tilde{\mathbf {x}}^T\left( \mathbf {X}^T\mathbf {X}\right) ^{-1}\tilde{\mathbf {x}}}}. \end{aligned}$$

Nonetheless, we note that to satisfy this constraint the tail decay of such a prior might be too rapid and an excessive amount of prior information might be included in the model, whereas the GIG distribution provides useful tools to control it and to specify a more suitable prior distribution.

### The Random Intercepts Model

The constraints on $\gamma ^2_{\tau ,s}$ that appear in condition (*ii*) of Theorem 1 look rather complicated as we assumed a general structure for $\mathbf {Z}$. To better understand the meaning of the result, we can show the results obtained when $\mathbf {Z}$ is simpler. Let us consider the following simple random intercept model, that can be applied in the analysis of repeated measurement data where a random effect is introduced to account for within individual correlation:

9 $$\begin{aligned} \begin{aligned} w_{ij}&=\log \left( y_{ij}\right) = \mathbf {x}_{ij}^T\varvec{\beta } +v_j +\varepsilon _{ij};\ j=1,...,m;\ i=1,...,n_j;\\&\varepsilon _{ij}|\sigma ^2 {\mathop {\sim }\limits ^{ind}} N\left( 0,\sigma ^2\right) ,\ v_j|\tau ^2 {\mathop {\sim }\limits ^{ind}} N\left( 0,\tau ^2\right) . \end{aligned} \end{aligned}$$

In the random intercepts model, the number *m* of the columns of $\mathbf {Z}$ coincides with the number of clusters observed in the data and each row contains a single 1, denoting that the correspondent unit belongs to the cluster (typically the subject in longitudinal data), and 0s otherwise. Moreover, $\mathbf {X}_o$ is the simple design matrix, since the first column is the usual $\varvec{1}_n$ vector corresponding to the general intercept and the first element of $\mathbf {x}_{o,i}$ is 1. Moreover, it is easy to verify that $l=p-rank\{\mathbf {X}^T\left( \mathbf {I}-\mathbf {P_Z} \right) \mathbf {X}\}=1$ and therefore the unique non-null entry of $\mathbf {L}_s$ is the first element of the first column. Eventually, exploiting the particular structure of $\mathbf {Z}$, after some algebra, it is possible to verify that $\mathbf {L}_{s;1,1}=m^{-1}$ (i.e., the inverse of the number of groups determined by $\mathbf {Z}$). Provided that priors (7) and (8) are adopted, the condition on $\gamma ^2_\sigma$ does not change, whereas the eventual condition on $\gamma ^2_{\tau }$ simplifies to:

$$\begin{aligned} \gamma ^2_{\tau } > r + \frac{r^2}{m}. \end{aligned}$$

## Practical Implementation Issues

In this section, we consider two issues related to practical implementation. In Sect. 4.1, we consider how to set GIG priors’ hyper-parameters. Theorem 1 provides lower bounds for the $\gamma$ parameters; we complement this information offering some guidance on how to remove the dependence on specific $\tilde{\mathbf {x}}$ in the choice of $\gamma$ and on how to choose values for $\lambda$ and $\delta$ parameters. The setting of these parameters can be relevant in the analysis of small samples. Specifically, we devise a weakly informative strategy based on the uniform shrinkage principle that will lead us to the specification of Gamma priors on the variance components.

In Section 4.2, we provide some details on how to generate samples from the posterior of model parameters (and the random effects). We only need a direct Gibbs sampler where elementary samplers can be used for each of the full conditionals: a nice feature that depends on the conjugacy relationship between the normal and the GIG distributions. To encourage the use of the method by practitioners and automatically set the advised priors, functions included in the BayesLN package can be used (Gardini et al., 2020).

### Hyper-Parameters Choice

The lower bounds in Theorem 1 depend on *r*, the order of posterior moments for which we need to impose the existence. In principle, a priori we would set $\gamma$s to the lower bound allowing the existence of moments up to the order *r* we are interested in, with the aim of avoiding priors with exceedingly light tails. In practice, it is advisable to set $\gamma$ parameters somewhat larger than the existence lower bound to avoid numerical instability caused by dealing with integrals that although finite are very large. We can achieve this, for instance, by choosing values of the $\gamma$s allowing the existence of moments up to the order $r+c$, with $c>0$. A discussion on the selection of *c* can be found in Section S1 in the supplementary material. In short, choices of $c \ge 0.5$ are advisable. Throughout the simulations and applications of this paper, we will use $c=1$.

The existence conditions stated in Theorem 1 also depend on $\tilde{\mathbf {x}}$ through $\tilde{\mathbf {x}}^T\left( \mathbf {X}^T\mathbf {X}\right) ^{-1}\tilde{\mathbf {x}}$. Since we want moments of order *r* to exist for all the $\tilde{\mathbf {x}}$ included in the analysis, the dependence on $\tilde{\mathbf {x}}$ can be removed by setting:

$$\begin{aligned} \gamma _\sigma =\sqrt{(r+c)+(r+c)^2h_m}, \end{aligned}$$

with $h_m=\max _{i \in s_p} \tilde{\mathbf {x}}_i^T\left( \mathbf {X}^T\mathbf {X}\right) ^{-1}\tilde{\mathbf {x}}_i$ where $s_p$ is the set of points in the covariates’s space for which we are interested in making predictions. If the moments of the posterior predictive distribution are required, then $(r+c)^2$ must be included in the previous condition. In the same line, we propose to set:

$$\begin{aligned} \gamma _{\tau ,s}=\sqrt{(r+c)+(r+c)^2l_m}, \end{aligned}$$

where $$l_m=\max _{i \in s_p} \tilde{\mathbf {x}}_{o,i}^T\mathbf {L}_s\tilde{\mathbf {x}}_{o_i}$$.

In general, the advice is to fix the parameter $\gamma$ equal to the most restrictive condition (i.e., the greatest one) with respect to the quantities that are of interest in the analysis.

As expected, constraints on the existence of posterior moments lead to priors with light tails for the variance components. In order to avoid excessively informative priors, we propose a weakly informative strategy for the selection of remaining parameters. To illustrate our heuristic, let us work on the notable special case where $q=1$. Consequently, for simplicity, we denote with $\tau ^2$ the variance component associated with the unique random effect. Some remarks on the generalization to the case $q>1$ are reported later. Let the intraclass correlation coefficient be defined as:

10 $$\begin{aligned} \rho =\frac{\tau ^2}{\sigma ^2+\tau ^2}. \end{aligned}$$

This quantity is of interest in the analysis of hierarchical model, both from a statistical viewpoint and from the applied perspective. Chaloner (1987) proposes to specify $\rho \sim \mathcal {U}(0,1)$ to obtain good frequentist properties for the parameters estimates. The uniform prior distribution for $\rho$ has been extensively studied and used (Daniels, 1999). If both variance components $\sigma ^2$ and $\tau ^2$ are GIG distributed, Favaro et al. (2012) show that $\rho$ follows a normalized generalized inverse Gaussian distribution, i.e., $\rho \sim N-GIG(\lambda _\tau ,\delta _\tau ,\gamma _\tau ,\lambda _\sigma ,\delta _\sigma , \gamma _\sigma )$. If we assume, for the time being, to set the same hyper-parameters for both priors, i.e., $\sigma ^2 \sim GIG(\lambda ,\delta ,\gamma ) \, and \, \tau ^2 \sim GIG(\lambda ,\delta ,\gamma )$, then the normalized GIG density for $\rho$ simplifies to:

11 $$\begin{aligned} p(\rho )=\frac{K_{2\lambda }\left( \gamma ^2\delta ^2\left[ \frac{1}{\rho }+\frac{1}{1-\rho }\right] \right) }{2\left[ K_\lambda \left( \gamma \delta \right) \right] }\left[ \rho (1-\rho )\right] ^{\lambda -1},\quad \rho \in (0,1). \end{aligned}$$

Moreover, considering the target functionals of the analysis, the most restrictive threshold should be chosen as the value of $\gamma$.

The resulting density is a function of the product $\delta \gamma$. To simplify the parameter specification, we consider the special case $\delta \rightarrow 0$ that frees the distribution from the dependence on both parameters and that makes the choice of different $\gamma$s due to different constraining equations immaterial for $p(\rho )$.

When $\delta \rightarrow 0$, the density (11) can be simplified further by using a small argument approximation to the Bessel *K* function:

$$\begin{aligned} p(\rho )\simeq \frac{\Gamma (|2\lambda |)}{\Gamma (|\lambda |)^2}\left[ \rho (1-\rho )\right] ^{|\lambda |-1},\quad \rho \in (0,1). \end{aligned}$$

Setting $\lambda =1$ implies $\rho \sim \mathcal {U} (0,1)$. If we consider $\phi =\frac{\tau ^2}{\sigma ^2}$, a one-to-one transformation of $\rho$, the prior implied by the above choices is $p(\phi )=(1+\phi ^2)^{-1}$, that is the solution proposed for $\phi$ by Ye (1994) within the reference prior framework (Berger and Bernardo, 1992).

The strategy can be summarized as:

$$\begin{aligned} \sigma ^2\sim GIG\left( \lambda =1, \delta =\varepsilon , \gamma _m\right) ,\quad \tau ^2\sim GIG\left( \lambda =1, \delta =\varepsilon , \gamma _m\right) ; \end{aligned}$$

where $\gamma _m$ is the most restrictive existence conditions for the considered quantities and $\varepsilon$ is some small constant close to 0 (e.g., 0.01). This proposal can be straightforwardly extended to the case $q>1$ assuming that a uniform prior is specified for every $\rho _s=\tau _s(\tau _s+\sigma ^2)^{-1}$. These marginal priors are retrieved setting all the priors on $\tau _s$ as independent and equal GIG distributions with parameters fixed according to the described strategy; i.e., $\tau ^2_s\sim GIG(\lambda =1, \delta =\varepsilon , \gamma _m),\ \forall s$.

We note that under the described setting, if the $\lambda$ parameter is set to be positive, a gamma prior $\mathcal {G}\left( \lambda ,\gamma ^2/2\right)$ for each variance component is approximately assumed. As a consequence, a normal-gamma prior is specified marginally for the random effects vector $\mathbf {u}$. This prior setting is not new to the literature as it was introduced by Griffin and Brown (2010) as prior for the coefficients of a linear model. Frühwirth-Schnatter and Wagner (2011) and Fabrizi et al. (2018) already use this distribution as prior for random intercepts. They note that these priors encourage shrinkage of the random intercepts toward the general intercept and more so as $\lambda$ gets smaller. If $\lambda =1$, the gamma distribution degenerates to the exponential distribution, and in that case the normal-gamma is a Laplace distribution. This particular prior is known also as *Bayesian Lasso* and is characterized by a spike in 0. In general, the degree of shrinkage determined by the prior can be increased setting $\lambda$ near 0, whereas increasing this parameter has an opposite effect.

The main difference between Griffin and Brown (2010), Frühwirth-Schnatter and Wagner (2011), and the present proposal is represented by the approach used to deal with the scale (or rate) parameter of the gamma prior. In fact, the cited papers specify an hyper-prior on it. This solution is not viable here because of the restrictions on the parameter space due to the posterior moments existence condition.

### Computational Algorithms

An appealing characteristic of the adoption of GIG priors (7) and (8) for the variance components of model (1) is their conditional conjugacy. This can be exploited to derive easy to sample full conditionals for the model parameters in order to implement a Gibbs sampler algorithm^1^ able to generate random samples from their posterior distributions:

12 $$\begin{aligned}&\sigma ^2|\varvec{\beta },\mathbf {u}, \varvec{\tau }^2,\mathbf {w}\sim GIG\left( \lambda _\sigma -\frac{n}{2}, \sqrt{\left( \mathbf {w}-\mathbf {X}\varvec{\beta }-\mathbf {Zu}\right) ^{T}\left( \mathbf {w}-\mathbf {X}\varvec{\beta }-\mathbf {Zu}\right) +\delta _\sigma ^2}, \gamma _\sigma \right) ; \end{aligned}$$

13 $$\begin{aligned}&\tau _s^2|\varvec{\beta },\mathbf {u}, \sigma ^2,\varvec{\tau }^2_{-s},\mathbf {w}\sim GIG \left( \lambda _{\tau , s}-\frac{m_s}{2}, \sqrt{\mathbf {u}_s^T\mathbf {u}_s+\delta _{\tau ,s}^2}, \gamma _{\tau ,s}\right) ,\qquad s=1,...,q; \end{aligned}$$

14 $$\begin{aligned}&\mathbf {u}|\varvec{\beta },\sigma ^2, \varvec{\tau }^2,\mathbf {w}\sim \mathcal {N}_{m}\left( \mathbf {V}_\mathbf {u}\mathbf {Z}^T\left( \mathbf {w}-\mathbf {X}\varvec{\beta }\right) ,\sigma ^2 \mathbf {V}_\mathbf {u}\right) ; \end{aligned}$$

15 $$\begin{aligned}&\varvec{\beta }|\mathbf {u},\sigma ^2, \varvec{\tau }^2,\mathbf {w}\sim \mathcal {N}_p\left( \left( \mathbf {X}^T\mathbf {X}\right) ^{-1}\mathbf {X}^T\left( \mathbf {w}-\mathbf {Zu}\right) , \sigma ^2\left( \mathbf {X}^T\mathbf {X}\right) ^{-1}\right) ; \end{aligned}$$

where $\mathbf {V}_\mathbf {u}=\left( \mathbf {Z}^T\mathbf {Z}+\sigma ^2\mathbf {D}^{-1}\right) ^{-1}$. The sampler has been implemented in C++ within the function LN_hierarchical() in the R package BayesLN.

## Simulations

In this section, we present two simulation exercises focused on simple models specified on the logarithm of the response variable. In the first place, we consider a special case of (9) where $\mathbf {x}_{ij}^T\varvec{\beta }=\mu$, that is a one-way ANOVA model. The aim is to assess the frequentist properties of posterior means as predictors of $\theta _m=\exp \left\{ \mu +\frac{\tau ^2+\sigma ^2}{2}\right\}$ and $\theta _{c}(v_j)=\exp \{\mu +v_j+\frac{\tau ^2}{2}\}$ under different choices for the priors $p(\sigma ^2)$, $p(\tau ^2)$. We also include summaries of the posterior of $\theta _m$ and $\theta _{c}(v_j)$ conditional on the variance components, i.e., treating the variances as known, as benchmarks. We devote special attention to the analysis of small samples, where the impact of the priors is more apparent. A second simulation exercise, with a data generating process characterized by the presence of a continuous covariate, aims at assessing the impact of alternative prior choices on the posterior distribution of regression coefficients and posterior predictive distributions. Details about this second simulation exercise are presented in Section S4 of the supplementary material.

In the first simulation exercise, we generate $B=2000$ samples from model (9) assuming $\mathbf {x}_{ij}^T \varvec{\beta } = \mu$ under 24 different scenarios obtained crossing the following choices for the parameters: $n_j=(2,5)$, $m=10$, $\phi =\tau ^2/\sigma ^2=(0.5,1,2)$ and $\sigma ^2=(0.05,0.25,0.5,0.75)$. The general mean in the logarithmic scale is set to 0, i.e., $\mu =0$. The considered grid of values for $\tau ^2$ and $\sigma ^2$ is aimed at covering the range log-scale variances most common in applications. The estimates that require Monte Carlo methods are based on 4000 iterations, after the first 1000 iterations are discarded as burn-in. The point predictors we compare are:

(i) The posterior means of $\theta _m$ and $\theta _c(v_j)$ when priors are:
  16 $$\begin{aligned} p(\mu )\propto 1,\ \ \sigma ^2\sim GIG\left( 1, 0.01, \gamma _{\text {m}}\right) ,\ \tau ^2\sim GIG\left( 1, 0.01, \gamma _{\text {m}}\right) , \end{aligned}$$
  where $\gamma _{\text {m}}=\max \{\gamma _\sigma , \gamma _{\tau ,1}\}= \sqrt{3+3^2m^{-1}}$, according to the suggestions provided in Sect. 4.1 in order to assure the posterior variance existence. The predictors will be denoted as $\hat{\theta }_m^{GIG}$ and $\hat{\theta }^{GIG}_c(v_j)$, and the function LN_hierarchical of the BayesLN package is used to estimate the model;
(ii) The posterior means of $\theta _m$ and $\theta _c(v_j)$ when priors are:
  17 $$\begin{aligned} p(\mu )\propto 1, \ \ \sigma ^2\sim IG(1,1),\ \ \tau ^2\sim IG(1,1), \end{aligned}$$
  that will be labeled as $\hat{\theta }_m^{IG}$ and $\hat{\theta }^{IG}_c(v_j)$. These priors for the variance components are suggested as default choice in the BANOVA package (Wedel and Dong, 2020). The algorithm for sampling from the posterior distributions is implemented in Stan (Carpenter et al., 2017);
(iii) The posterior means of $\theta _m$ and $\theta _c(v_j)$ under small parameters inverse gamma (“Jeffreys like”) priors (Carpenter et al., 2018a):
  18 $$\begin{aligned} p(\mu )\propto 1, \ \ \sigma ^2\sim IG(0.001,0.001),\ \ \tau ^2\sim IG(0.001,0.001), \end{aligned}$$
  that will be labeled as $\hat{\theta }_m^{J}$ and $\hat{\theta }^{J}_c(v_j)$. The algorithm for sampling from the posterior distributions is implemented in Stan. An alternative choice of the IG parameters and namely $\sigma ^2\sim IG(1,0.001)$ and $\tau ^2\sim IG(1,0.001)$ is also considered. For brevity, results related to these latter alternatives are reported in section S3 of the supplementary material;
(iv) A *conditional* Bayes predictors in which $\sigma ^2$ and $\tau ^2$ are assumed to be known for the case of $\theta _m$ prediction:
  19 $$\begin{aligned} \hat{\theta }_m^{c}=\exp \left\{ \bar{w}+\frac{\sigma ^2+\tau ^2}{2}-\frac{3\left( \sigma ^2+n_g\tau ^2\right) }{2n} \right\} . \end{aligned}$$
  In line with Zellner (1971), we can show that (19) reaches minimum frequentist MSE among the predictors of $\theta _m$ having form $k\exp {\{\bar{w}\}}$. For benchmarking purposes, a minimum MSE estimator conditioned to the variance components for the functional $\theta _c(\nu _j)$ is useful too. In this case, a decision to take is the estimator class, since the global sample mean $\bar{w}$ as the only argument of the exponential function appears to be not appropriated. A heuristic strategy to obtain a conditioned estimator might be based on the derivation of the Bayes estimator under relative quadratic loss, obtaining:
  20 $$\begin{aligned} \hat{\theta }_{c}^{c}\left( v_j\right) =\exp \left\{ \frac{\sigma ^2}{\sigma ^2+n_g\tau ^2}\left( \frac{\tau ^2n_g}{\sigma ^2}\bar{w}_{.j}-\bar{w}\right) +\frac{\sigma ^2}{2}-\frac{3}{2}\frac{\sigma ^2}{\sigma ^2+n_g\tau ^2}\left( \tau ^2+\frac{\sigma ^2}{n}\right) \right\} . \end{aligned}$$
  The derivations of these estimators can be found in Section S2 of online supplementary material^2^.

Bias, root mean square error (RMSE), frequentist coverage and average interval width are reported for estimators of $\theta _m$ (for which we use the generic notation $\hat{\theta }_m$). Specifically, we calculate:

$$\begin{aligned} Bias\left( \hat{\theta }_m\right)&=\frac{1}{B}\sum _{k=1}^B \left( \hat{\theta }_m^{(k)}-\theta _m\right) ;\ RMSE(\hat{\theta }_m)=\sqrt{\frac{1}{B}\sum _{k=1}^B \left( \hat{\theta }_m^{(k)}-\theta _m\right) ^2 };\\ Cov\left( \hat{\theta }_m\right)&=\frac{1}{B}\sum _{k=1}^B \varvec{1}_{\left[ \hat{L}^{(k)};\hat{U}^{(k)}\right] }\left( \theta _m^{(k)}\right) ;\ Wid\left( \hat{\theta }_m\right) =\frac{1}{B}\sum _{k=1}^B \left( \hat{U}^{(k)}-\hat{L}^{(k)}\right) ; \end{aligned}$$

where $\hat{L}^{(k)}$ and $\hat{U}^{(k)}$ are computed as the 0.025 and 0.975 quantiles of the posterior distributions in question. In these formulas, $\hat{\theta }_m^{(k)}$ is the estimate of the true overall expectation $\theta _m$ at Monte Carlo iteration *k* and $\hat{L}^{(k)}$ and $\hat{U}^{(k)}$ are the estimated lower bound and upper bound for the $95\%$ intervals.

To jointly evaluate the *m* different estimates for $\theta _c(v_j),\ j=1,...,m$, an average evaluation of the estimates, that we denote with $\hat{\bar{\theta }}_c$, is required. Therefore, the relative absolute bias (RABias), the relative RMSE (RRMSE), the average frequentist coverage (ACo.) and the average interval width (AWi.) are studied.

More in detail we define the quantities:

$$\begin{aligned} RABias\left( \hat{\bar{\theta }}_c\right)&=\frac{1}{J}\sum _{j=1}^J\left| \frac{1}{B}\sum _{k=1}^B \left( \frac{\hat{\theta }_c^{(k)}\left( v_j\right) -\theta _c^{(k)}\left( v_j\right) }{\theta _c^{(k)}\left( v_j\right) }\right) \right| ;\\ RRMSE\left( \hat{\bar{\theta }}_c\right)&=\frac{1}{J}\sum _{j=1}^J\sqrt{\frac{1}{B}\sum _{k=1}^B \left( \frac{\hat{\theta }_c^{(k)}\left( v_j\right) -\theta _c^{(k)}\left( v_j\right) }{\theta _c^{(k)}\left( v_j\right) }\right) ^2 };\\ ACo\left( \hat{\bar{\theta }}_c\right)&=\frac{1}{J}\sum _{j=1}^J\frac{1}{B}\sum _{k=1}^B \varvec{1}_{\left[ \hat{L}^{(k)}\left( v_j\right) ;\hat{U}^{(k)}\left( v_j\right) \right] }\left( \theta _c^{(k)}\left( v_j\right) \right) ;\\ AWi\left( \hat{\bar{\theta }}_c\right)&=\frac{1}{J}\sum _{j=1}^J\frac{1}{B}\sum _{k=1}^B \left( \hat{U}^{(k)}\left( v_j\right) -\hat{L}^{(k)}\left( v_j\right) \right) ; \end{aligned}$$

where $\hat{L}^{(k)}(v_j)$ and $\hat{L}^{(k)}(v_j)$ are calculated as the 0.025 and 0.975 percentiles of the posterior distributions and $\hat{\theta }_c^{(k)}(v_j)$ is the estimate of the *j*-th true group specific expectation $\theta _c^{(k)}(v_j)$ at Monte Carlo iteration *k*.

In Tables 1 and S1 (the latter in Section S3 of the online supplementary material), we can see the frequentist properties of the point estimators of $\theta _m$: problems occurring to posterior means under inverse gamma priors for variance components ($\theta ^{IG}_m$ and $\theta ^{J}_m$) are apparent. In fact, extremely high values for bias and RMSE are detected. These anomalies can be considered as the numerical equivalent of the analytical non-finiteness of posterior moments. On the other hand, under our proposed prior, the estimators reach RMSE values that keep the same magnitude of the ones obtained for the benchmark $\theta _m^c$, showing their reliability.

Moving to results about group means (Tables 2 and S2), we note that observing numerically the analytical problems proved for $\theta _c^{IG}(v_j)$ and $\theta _c^{J}(v_j)$ is harder. In these cases, explosive numerical situations are not evident, even if we can say that our proposal $\theta _c^{GIG}(v_j)$ systematically outperforms the other considered estimators.

**Table 1:** Bias and RMSE for the considered estimators of $\theta _m$ in the different scenarios with $n_g=2$.

			$\theta ^{c}_m$		$\theta ^{IG}_m$		$\theta ^{J}_m$		$\theta ^{GIG}_m$	
$\phi$	$\sigma ^2$	$\theta _m$	Bias	RMSE	Bias	RMSE	Bias	RMSE	Bias	RMSE
0.5	0.05	1.038	− 0.005	0.073	0.305	0.320	0.015	0.078	0.034	0.087
	0.25	1.206	− 0.030	0.189	0.437	0.534	0.100	0.271	0.139	0.281
	0.5	1.455	− 0.072	0.320	0.907	6.543	2.408	77.027	0.241	0.515
	0.75	1.755	− 0.128	0.470	$>10^4$	$>10^4$	$>10^4$	$>10^4$	0.317	0.772
1	0.05	1.051	− 0.008	0.092	0.309	0.332	0.022	0.101	0.044	0.111
	0.25	1.284	− 0.047	0.248	0.644	4.943	0.241	2.113	0.173	0.373
	0.5	1.649	− 0.119	0.446	52.387	$>10^4$	$>10^4$	$>10^4$	0.284	0.711
	0.75	2.117	− 0.225	0.694	$>10^4$	$>10^4$	$>10^4$	$>10^4$	0.349	1.110
2	0.05	1.078	− 0.013	0.122	0.319	0.360	0.038	0.143	0.063	0.155
	0.25	1.455	− 0.087	0.364	7.232	285.148	$>10^4$	$>10^4$	0.224	0.556
	0.5	2.117	− 0.246	0.737	$>10^4$	$>10^4$	$>10^4$	$>10^4$	0.314	1.143
	0.75	3.08	− 0.519	1.291	$>10^4$	$>10^4$	$>10^4$	$>10^4$	0.253	1.942

In the supplementary material, results about the frequentist properties of credible intervals are reported for $\theta _m$ (Table S3) and averaged for the group specific expectations (Table S4). Considering both the inferential problems, we can summarize the results as follows: under all priors, systematic deviations from the nominal coverage level of 0.95 are not evident. Considering the intervals width, it emerges that the ones produced under GIG priors are almost always narrower than intervals produced under inverse gamma priors. This is particularly evident in the case of $\theta _m$. In particular, larger intervals are obtained under *IG*(1, 1) prior for variance components: probably it is not an appropriate choice in cases of variance components near to 0, as often happens in log-transformed data.

**Table 2:** RABias and RRMSE for the considered estimators of the group-specific expectations in the different scenarios with $n_g=2$.

		$\theta ^{c}_c(v_j)$		$\theta ^{IG}_c(v_j)$		$\theta ^{J}_c(v_j)$		$\theta ^{GIG}_c(v_j)$	
$\phi$	$\sigma ^2$	RABias	RRMSE	RABias	RRMSE	RABias	RRMSE	RABias	RRMSE
0.5	0.05	0.014	0.117	0.132	0.196	0.019	0.128	0.024	0.128
	0.25	0.067	0.257	0.192	0.391	0.113	0.349	0.109	0.329
	0.5	0.130	0.357	0.282	0.601	0.260	0.629	0.198	0.529
	0.75	0.188	0.430	0.394	0.836	0.458	1.002	0.273	0.712
1	0.05	0.018	0.131	0.136	0.204	0.027	0.151	0.030	0.144
	0.25	0.085	0.288	0.217	0.433	0.163	0.446	0.143	0.394
	0.5	0.163	0.398	0.348	0.714	0.395	0.892	0.272	0.668
	0.75	0.233	0.476	0.525	1.074	0.798	2.787	0.394	0.951
2	0.05	0.021	0.141	0.144	0.218	0.035	0.169	0.038	0.160
	0.25	0.099	0.309	0.258	0.499	0.214	0.542	0.183	0.464
	0.5	0.188	0.426	0.452	0.895	0.564	1.320	0.364	0.844
	0.75	0.268	0.509	0.757	1.593	1.739	19.881	0.550	1.286

In Section S3 of the supplementary material, results about this simulation setting under three further prior settings are presented. The first two explore the sensitivity of posterior with respect to different choices of the GIG scale parameter $\delta$. Specifically, we consider the settings $\delta =0.1$ and $\delta =0.001$. It is interesting to note that we obtain results extremely close to those under prior (16). The third simulation setting involves the alternative choice for the IG hyper-parameters described below formula (18). Results point in the direction of non-existence of posterior moments showing also issues in the estimation of the group means.

As far as the second simulation exercise, we mentioned above is concerned, the results (reported in Section S4 of the supplementary material) show that different priors on the variance components do not induce remarkable changes on the estimation of a regression coefficient, whereas the problems affecting the moments of $\theta _m$ and $\theta _c(v_j)$ emerges also for the posterior predictive distribution, in line with theoretical findings.

## Real Data Application: Reading Times

Several applications in psychology and cognitive sciences have as central output the time requested to perform some tasks. By definition, times are positive numbers and often show a positively skewed distribution: for these reasons, it is common to analyze their logarithmic transformations.

The data we use to apply our methodologies were originally collected by Gibson and Wu (2013) in order to investigate the presence of a notable difference between times requested to process a subject-extracted relative clause (SRC) and an object-extracted relative clause (ORC) in Chinese language. In particular, times (in milliseconds) required to read the head noun of a Chinese clause are registered under a repeated measure design characterized by two factors: subject and reading item.

This dataset has been analyzed also by Sorensen and Vasishth (2015), that proposed a Bayesian linear mixed model specified for the reading time logarithm. Here, we consider the model formulation with two random intercepts related to the grouping factors:

$$\begin{aligned} w_{ijk}=\log \left( y_{ijk}\right) =\beta _0+\beta _1 x_{i}+u_j+v_k+\varepsilon _{ijk}, \end{aligned}$$

where $y_{ijk}$ is the reading time observed for subject $j=1,...,37$, reading item $k=1,...,15$ and clause type $i=1,2$. More in detail, it is fixed $x_i=-1$ in case of SRC, and $x_i=1$ for ORC. The random effects are aimed at accounting for the potential within subject and within item correlation, and they are assumed to be independently distributed as $u_j|\tau _u^2\sim \mathcal {N}\left( 0,\tau _u^2\right)$ and $v_k|\tau _v^2\sim \mathcal {N}\left( 0,\tau _v^2\right)$. Both of them are assumed independent from the error $\varepsilon _{ijk}|\sigma ^2\sim \mathcal {N}\left( 0,\sigma ^2\right)$. Beyond the usual inference on the model parameters that are related to times in the log-scale, to have a clearer interpretation of the studied phenomenon the estimation and prediction of quantities in the original data scale might be relevant. For example, the expectation conditioned on clause type and marginalized with respect both the random effects:

$$\begin{aligned} \theta _m\left( x_i=\pm 1\right) =\exp \left\{ \beta _0\pm \beta _1+\frac{\tau ^2_u+\tau ^2_v+\sigma ^2}{2} \right\} . \end{aligned}$$

On the other hand, the expectation specific of a particular subject and item (individual) is:

$$\begin{aligned} \theta _c\left( x_i,u_j,v_k\right) =\exp \left\{ \beta _0+x_i\beta _1+u_j+v_k+\frac{\sigma ^2}{2} \right\} . \end{aligned}$$

From an interpretative viewpoint, it can be useful to target the expected time conditioned to only a particular random effect, e.g., integrating out only the subject and considering only a particular item:

$$\begin{aligned} \theta _c\left( x_i,v_k\right) =\exp \left\{ \beta _0+x_i\beta _1+v_k+\frac{\tau _u^2+\sigma ^2}{2} \right\} . \end{aligned}$$

Obtaining posterior summaries of these functionals might help in understanding the phenomenon and communicating results.

More technically, the design matrix $\mathbf {Z}$ for the random effects is constituted by two blocks, in order to define two distinct random intercepts: $\mathbf {Z}=\left[ \mathbf {Z}_v\ \ \mathbf {Z}_u \right]$. The elements of $\mathbf {Z}_v\in \mathbb {R}^{n\times 15}$ assume value 1 in column *k* if the observation is related to the item *k* and 0 otherwise; on the other hand, $\mathbf {Z}_u\in \mathbb {R}^{n\times 37}$ assume value 1 in column *j* if the observation is related to subject *j* and 0 otherwise.

As a consequence, the rank deficiency of $\mathbf {X}\left( \mathbf {I}-\mathbf {P}_Z \right) \mathbf {X}$ is $l=1$ and it is due to the fixed effect intercept, which is linearly dependent with respect to both $\mathbf {Z}_v$ and $\mathbf {Z}_u$.

Hyper-parameters $\gamma$ in priors (7) and (8) are set along the lines of Section 4.1 in order to assure the existence of the first two posterior moments. For $\sigma ^2$, we apply condition (*i*) in Theorem 1 by setting $r=3$ for numerical stability and calculating the maximum leverage: we obtain $\gamma _{\sigma }=1.742$. For the random effects variances, $\mathbf {L}_v\in \mathbb {R}^{2\times 2}$ and $\mathbf {L}_u\in \mathbb {R}^{2\times 2}$ must be computed, whereas $\mathbf {X}_o$ coincides with $\mathbf {X}$ since the rank deficiency is due to the intercept. Given that $l=1$, the unique non-null elements coincide with the inverse of the first elements of the matrices $\mathbf {X}^T\left( \mathbf {Z}(\mathbf {Z}^T\mathbf {Z})^{-}\mathbf {C}_v (\mathbf {Z}^T\mathbf {Z})^{-}\mathbf {Z}^T\right) \mathbf {X}$ and $\mathbf {X}^T\left( \mathbf {Z}(\mathbf {Z}^T\mathbf {Z})^{-}\mathbf {C}_u (\mathbf {Z}^T\mathbf {Z})^{-}\mathbf {Z}^T\right) \mathbf {X}$, where $\mathbf {C}_v=\text {diag}\left( \mathbf {I}_{15}, \varvec{0}_{37}\right)$ and $\mathbf {C}_u=\text {diag}\left( \varvec{0}_{15}, \mathbf {I}_{37}\right)$. The deduced numerical conditions are $\gamma _{\tau ,v}=2.046$ and $\gamma _{\tau ,v}=2.434$; therefore, the latter value is chosen for all the GIG priors tail parameters since it is the more restrictive condition. We stress that the available package BayesLN (Gardini et al., 2020) automatically produces these computations to facilitate the usage by practitioners. The code required to obtain the results presented in this section is available as supplementary material, whereas details on the MCMC convergence diagnostics are reported in Section S5 of the supplementary material.

**Table 3:** Posterior means and standard deviations obtained for the whole dataset ($n=547$) under three considered prior specifications.

	$GIG(1,0.01,\gamma )$		*IG*(1, 1)		*IG*(0.001, 0.001)	
	Mean	SD	Mean	SD	Mean	SD
$\tau ^2_u$	0.068	0.022	0.131	0.034	0.063	0.020
$\tau ^2_v$	0.046	0.026	0.185	0.074	0.038	0.020
$\sigma ^2$	0.270	0.017	0.271	0.017	0.270	0.017
$\beta _0$	6.060	0.073	6.062	0.124	6.062	0.068
$\beta _1$	− 0.036	0.022	− 0.035	0.022	− 0.036	0.022
$\theta _m(x_i=- 1)$	539.351	43.168	601.487	81.673	536.936	39.894
$\theta _m(x_i=1)$	502.195	40.032	560.208	75.651	499.748	36.931
$\theta _c(-1,u_{3})$	514.441	48.332	530.301	57.406	513.261	47.800
$\theta _c(1,u_3)$	479.001	44.836	493.922	53.165	477.721	44.418

In Table 3, the posterior means and standard deviations obtained for the complete dataset ($n=547$) under prior settings (16), (17) and (18) are reported. Posterior inference has been carried out both on basic model parameters and some conditional expectations of reading times. In particular, $\theta _m(x_i=- 1)$ represents the expected time requested to process a SRC estimated by the model, whereas $\theta _m(x_i=1)$ is the time expected for an ORC. Another interesting output for these kind of models is the estimation of the response variable expectation within a particular group: for example, $\theta _c(-1,u_{3})$ represents the average reading time for item $j=3$ in the SRC case and $\theta _c(1,u_{3})$ in the ORC case.

**Table 4:** Posterior means and standard deviations obtained for a subset of the dataset ($n=110$) under three considered prior specifications.

	$GIG(1,0.01,\gamma )$		*IG*(1, 1)		*IG*(0.001, 0.001)	
	Mean	SD	Mean	SD	Mean	SD
$\tau ^2_u$	0.062	0.024	0.154	0.043	0.064	0.030
$\tau ^2_v$	0.024	0.027	1.063	1.96	0.046	0.611
$\sigma ^2$	0.132	0.021	0.152	0.025	0.140	0.026
$\beta _0$	5.958	0.103	5.954	0.639	5.953	0.121
$\beta _1$	− 0.054	0.036	− 0.054	0.039	− 0.055	0.037
$\theta _m(x_i=- 1)$	458.242	52.045	$1.5\times 10^{11}$	$1.4\times 10^{13}$	$3.3\times 10^{8}$	$3.3\times 10^{10}$
$\theta _m(x_i=1)$	411.008	46.569	$1.4\times 10^{11}$	$1.4\times 10^{13}$	$3.7\times 10^{8}$	$3.7\times 10^{10}$
$\theta _c(-1,u_{3})$	475.965	38.465	512.621	53.087	475.136	39.349
$\theta _c(1,u_3)$	426.903	34.289	460.308	47.627	425.531	34.472

**Fig. 1:**
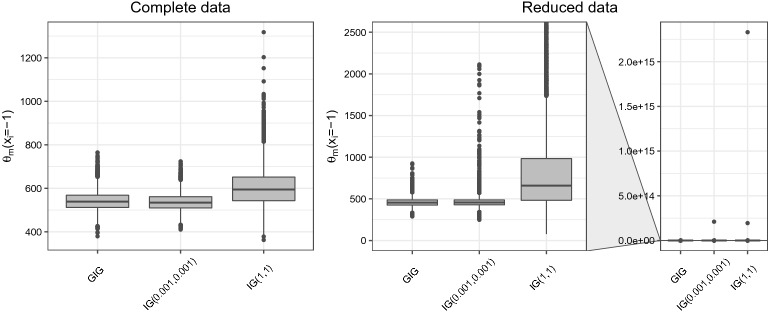
Posterior distributions of the marginal means $\theta _m(x_i=- 1)$ under different priors for the variance components. The results obtained with the complete and the reduced data are shown.

We note that the issues that affect posterior moments of functionals in the original data scale are masked by the moderately large sample size. In fact, there are no clear symptoms of the fact that posterior results obtained under inverse gamma priors are theoretically meaningless, since they are MCMC estimates of integrals that are analytically not finite, as already noted in the simulation section. We also note that the inverse gamma prior with parameters both equal to 1 can be a largely informative prior for variances when their actual value is near to 0, as it often happens in the analysis of log-transformed data. In this application, the variance components ($\tau ^2_u$ and $\tau ^2_v$) posterior estimates are substantially higher than the ones obtained under the proposed GIG priors and the small-parameters inverse gamma priors.

Finally, we fit the same model under the three prior settings on a subset of the original dataset: we considered reading time observations from the first three clauses only ($k=1,2,3$ and $n=110$). In Table 4, posterior results are displayed. The aim of this second exercise is to stress again the mathematical inconsistency of the conditional expectations posterior summaries in Table 3: we note that, in this case, the infiniteness of the target integrals is evident also from their MCMC estimates. The cause of this feature appears in Fig. 1 where the boxplots representing the posterior distribution of $\theta _m(x_i=-1)$ highlight the heavy tails obtained under IG priors for the reduced dataset. On the other hand, our prior specification allows to produce reliable estimates in any case, improving the readability of the log-normal mixed model results.

## Discussion

In this section, we discuss the scope of the methodology we introduced and its limitations. As noted in Sect. 2.1, model (1) does not include special cases in which random effects are correlated and the modelling of their dependence involves additional parameters.

Models with these features can be relevant in some applications, for instance when a random intercept and a random slope are specified within a single grouping factor (Sorensen and Vasishth, 2015; Jackman, 2009, Chapter 7). A complete coverage of models with correlated random effects is beyond the scope of this paper, in which we focused on analytically treatable models for which relevant posteriors can be explored using direct Gibbs sampling.

Nonetheless, in this section we study a simple model in which a vector of random intercepts $\mathbf {u}_0$ and random slopes $\mathbf {u}_1$ are included in the model (i.e., $q=2$). We assume that pairwise elements of these vectors refer to the same grouping factor with levels $j=1,\dots m$. For the *j*-th component $\mathbf {u}_{j}=\left( u_{0,j},u_{1,j}\right) ^T$, we assume the following distribution:

21 $$\begin{aligned} \mathbf {u}_{j}|\rho , \tau _{0}^2,\tau _{1}^2 \sim \mathcal {N}_2\left( \varvec{0}, \left[ \begin{matrix} \tau _{0}^2&{}\rho \tau _{0}\tau _{1}\\ \rho \tau _{0}\tau _{1}&{}\tau _{1}^2 \end{matrix}\right] \right) , \end{aligned}$$

where $\rho$ is the correlation parameter. The study of this case allows us to show that the results of Theorem 1 apply more generally than to model (1). We can state the following result:

### Corollary 1

The normal linear mixed model in the log scale

$$\begin{aligned} \mathbf {w}|\mathbf {u}, \varvec{\beta }, \sigma ^2\sim \mathcal {N}_n\left( \mathbf {X}\varvec{\beta }+\mathbf {Zu}, \mathbf {I}_n\sigma ^2 \right) \end{aligned}$$

is considered with $\mathbf {u}=\left[ \mathbf {u}_0^T, \mathbf {u}_1^T\right] ^T$ and

$$\begin{aligned} \mathbf {u}|\rho ,\tau ^2_0,\tau ^2_0\sim \mathcal {N}_{2m}\left( \mathbf {0}, \mathbf {D}\right) ,\ \mathbf {D}= \left[ \begin{matrix} \tau _0^2\mathbf {I}_{m} &{}\rho \tau _{0}\tau _{1}\mathbf {I}_{m}\\ \rho \tau _{0}\tau _{1}\mathbf {I}_{m}&{} \tau _1^2\mathbf {I}_{m}\\ \end{matrix}\right] . \end{aligned}$$

The priors (7) and (8) are assumed for the variance components, along with $\rho \sim \mathcal {U}\left( -1,1\right)$. In order to compute the *r*-th, with $r>0$, posterior moment of $\theta _c(\tilde{\mathbf {x}},\tilde{\mathbf {z}})$, $\theta _m(\tilde{\mathbf {x}})$ and of $p(\tilde{y}|\mathbf {y})$, the same constraints on the prior parameters as those derived in Theorem 1 must be imposed.

### Proof

See Section S6 in the Supplementary material. $\square$

The previous result allows to extend the existing conditions for moments of functional studied in Theorem 1 to models that considers several grouping factors determining this kind of correlated random effects. However we note that, introducing additional parameters to account for the correlation, a simple Gibbs sampler to draw from the parameters posterior cannot be used anymore. Nonetheless, models of this type can be easily fitted through platforms for statistical computation such as Stan. Specifically, as the GIG is not currently available among the pre-specified distributions in Stan, a function allowing the specification of such distribution as prior for the variance parameters is provided in Section S6.

The log is a special case of the Box-Cox family of transformations (Box and Cox, 1964). In many applications, the whole family is considered and the transformation ruling parameter, $\ell$, is chosen on the basis of the available sample, while, under the Bayesian approach, a prior distribution $p(\ell )$ needs to be specified in order to account for the uncertainty associated with its choice.

The log-transformation plays a central role among those of the Box-Cox family because of its popularity, the well-known properties of the log-normal distribution, and the fact that linear models on the log-scale are multiplicative on the original scale, a specification that is often appropriate in applied problems. The extension of our results to linear mixed models specified on Box-Cox transformed responses is beyond the scope of this paper since the inferential problem would be substantially different. In fact, an additional parameter $\ell$ would be involved and a prior distribution must specified or, more appropriately, a joint prior distribution for $\ell$, the variance components, and the slope coefficients, as suggested in Sweeting (1984).

Here, we simply note that, at least for predictive distributions, the non-existence of posterior moments is still an issue: De Oliveira et al. (1997), studying a Gaussian random fields that generalizes model (1) when $q=1$, note that, once a ordinary inverse Gamma distribution for the variance components is assumed, the expected value of the posterior predictive distribution is not finite whenever $-1\le \ell \le (n-p)^{-1}$.

Obtaining general results similar to those in Theorem 1 for the general Box-Cox transformation is difficult because of the complicated expressions that functionals similar to (3) and (4) have in the general case. Nonetheless, we note that the results stated for the suggested priors hold whenever $\ell > 0$ as the implied underlying distribution would have lighter tails than the log-normal.

## Conclusions

The use of linear mixed models on log-transformed response variables is widespread in several applied fields. In this paper, the model is investigated within the Bayesian framework. Inferential problems that arise when predicting response variable values and estimating its expectation in the original scale are pointed out. Specifically, the posterior distributions have not finite moments under the most common priors for the variance components. This would make simple posterior summaries based on popular loss functions such as the quadratic one, not valid. Following the results obtained in Theorem 1, the Generalized Inverse Gaussian distribution endowed with a careful choice of hyper-parameters is proposed as prior for the variance components in the model, to obtain posteriors with moments defined up to a pre-specified order.

We tried to provide all the tools needed by a practitioner to exploit the proposed methodology. In particular, the R package BayesLN contains the LN_hier_existence function that computes the existence conditions for the posterior moments derived in Theorem 1 and LN_hierarchical that allows to carry out posterior inference on model (1).

The paper covers the case of a linear mixed model multiple random effects assumed conditionally independent. This latter assumption, that can be restrictive in some applications, is motivated by the attempt to achieve a balance between model generality, analytical tractability, and computational ease of implementation. However, since in the behavioral sciences literature the need for specifying correlated random effects within a common grouping factor (e.g., random intercept and random slopes) can emerge, the extension of the main results to this case is also discussed. To help the practical implementation in this case, we provide Stan code useful to specify the proposed GIG priors, allowing to fit models that include correlated random effects.

## Supplementary material

In the supplementary material, the following information is reported. In Section S1, we complement the discussion on the choice of prior specification for the hyper-parameters $\gamma$ contained in Sect. 4.1 of the main paper. In Section S2, the minimum MSE estimator conditioned to the variance components of the overall mean $\theta _m$ is derived and its connection to the Bayesian framework is explained. This quantity is used as benchmark in the simulation study. In Section S3, some additional tables concerning the results of the simulation discussed in Section 5 of the paper are reported. Section S4 contains an additional simulation study in which covariates are included in the model, and the frequentist properties of the posterior predictive distribution are investigated. Section S5 reports the information about the convergence diagnostics of the MCMC algorithm used to fit the models compared in the application of Section 6. Eventually, the proof of Corollary 1 and some software details useful to estimate models with dependent random effects are contained in Section S6. All the R code used for the simulations and the application is available in a zipped folder.

### Supplementary Information

Below is the link to the electronic supplementary material.

Supplementary material 1 (pdf 845 KB)

## Appendix: Proof of Theorem 1

(*i*) The *r*-th moment of $\theta _c(\tilde{\mathbf {x}},\tilde{\mathbf {z}})$ can be defined as:

$$\begin{aligned} \begin{aligned} \mathbb {E}\left[ \theta _c^r\left( \tilde{\mathbf {x}},\tilde{\mathbf {z}}\right) |\mathbf {w}\right]&=\mathbb {E}\left[ \exp \left\{ r\tilde{\mathbf {x}}^T\varvec{\beta }+r\tilde{\mathbf {z}}^T\mathbf {u}+r\frac{\sigma ^2}{2}\right\} \big |\mathbf {w}\right] \\&=\int _{\varvec{\Theta }}\exp \left\{ r\tilde{\mathbf {x}}^T\varvec{\beta }+r\tilde{\mathbf {z}}^T\mathbf {u}+r\frac{\sigma ^2}{2}\right\} p(\varvec{\beta },\mathbf {u}, \sigma ^2, \varvec{\tau }^2|\mathbf {w})\mathrm {d}\varvec{\theta }. \end{aligned} \end{aligned}$$

Recalling the expression (4) and performing a simple change of variable, it is possible to solve the integral, twice recognizing the moment generating function of a Gaussian distribution: the first related to the $\mathcal {N}\left( \tilde{\mathbf {z}}^T\mathbf {V}_\mathbf {u}\mathbf {Z}^T\left( \mathbf {z}-\mathbf {X}\varvec{\beta }\right) ,\sigma ^2 \tilde{\mathbf {z}}^T\mathbf {V}_\mathbf {u}\tilde{\mathbf {z}}\right)$ and the second to $\mathcal {N}_p\left( \tilde{\mathbf {q}}^T\bar{\varvec{\beta }},\tilde{\mathbf {q}}^T\mathbf {V}_\beta \tilde{\mathbf {q}}\right)$, where $\tilde{\mathbf {q}}^T=\tilde{\mathbf {z}}^T\mathbf {V}_\mathbf {u}\mathbf {Z}^T\mathbf {X}-\tilde{\mathbf {x}}^T$ and $\mathbf {V}_{\mathbf {u}}=\Big (\mathbf {Z}^T\mathbf {Z} + \sigma ^2\mathbf {D}^{-1}\Big )^{-1}$. Then, the following integral is obtained:

$$\begin{aligned} \begin{aligned}&\int _{0}^{+\infty }\cdots \int _{0}^{+\infty }g(\sigma ^2,\varvec{\tau }^2)\exp \left\{ -\frac{1}{2} \sigma ^2\left( \gamma ^2_\sigma -r+\right. \right. \\&\quad \left. \left. \quad -r^2\left[ \tilde{\mathbf {z}}^T\mathbf {V}_\mathbf {u}\tilde{\mathbf {z}}+\frac{\tilde{\mathbf {q}}^T\mathbf {V}_\beta \tilde{\mathbf {q}}}{\sigma ^2}\right] \right) \right\} \mathrm {d}\varvec{\tau }^2 \mathrm {d}\sigma ^2, \end{aligned} \end{aligned}$$

where $g(\sigma ^2,\varvec{\tau }^2)$ is a function that does not affect the finiteness of the integral. Therefore, the integral is finite when:

$$\begin{aligned} \lim _{\sigma ^2\rightarrow +\infty }\left( \gamma ^2_\sigma -r-r^2\left[ \tilde{\mathbf {z}}^T\mathbf {V}_\mathbf {u}\tilde{\mathbf {z}}+\frac{\tilde{\mathbf {q}}^T\mathbf {V}_\beta \tilde{\mathbf {q}}}{\sigma ^2}\right] \right) >0. \end{aligned}$$

In order to compute this limit, lemma 1 by Hobert and Casella (1996) is useful. It states that, given a scalar *c* and a non-negative definite matrix $\mathbf {S}$, the limit:

A1 $$\begin{aligned} \lim _{c\rightarrow +\infty } \left( \mathbf {S}+\frac{\mathbf {I}}{c}\right) ^{-1} \end{aligned}$$

coincides with a generalized inverse of $\mathbf {S}$. Moreover, it is immediate to extend the result to the case in which any diagonal matrix substitutes $\mathbf {I}$.

Considering the limit of the factor that multiplies $r^2$ and focusing on the first addend, by applying the previous result and doing some computations, it is possible to show that

A2 $$\begin{aligned} \lim _{\sigma ^2\rightarrow +\infty } \tilde{\mathbf {z}}^T\left[ \mathbf {Z}^T\mathbf {Z}+\sigma ^2\mathbf {D}^{-1} \right] ^{-1} \tilde{\mathbf {z}}= \lim _{\sigma ^2\rightarrow +\infty }\frac{1}{\sigma ^2} \tilde{\mathbf {z}}^T\left[ \frac{\mathbf {Z}^T\mathbf {Z}}{\sigma ^2}+\mathbf {D}^{-1} \right] ^{-1} \tilde{\mathbf {z}} =0. \end{aligned}$$

Then, the limit of the second added must be computed. It is:

$$\begin{aligned} \lim _{\sigma ^2\rightarrow +\infty }\frac{\tilde{\mathbf {q}}^T\mathbf {V}_\beta \tilde{\mathbf {q}}}{\sigma ^2}=\lim _{\sigma ^2\rightarrow +\infty }\tilde{\mathbf {q}}^T\left( \mathbf {X}^T\mathbf {X}+\sigma ^2\mathbf {X}^T\mathbf {MX} \right) ^{-1}\tilde{\mathbf {q}}. \end{aligned}$$

Focusing on the structure of the matrix $\mathbf {M}$:

$$\begin{aligned} \sigma ^2\mathbf {X}^T\mathbf {MX}=\mathbf {X}^T\left( \mathbf {Z}(\mathbf {Z}^T\mathbf {Z})^{-} \left( (\mathbf {Z}^T\mathbf {Z})^{-}+\frac{\mathbf {D}}{\sigma ^2}\right) ^{-1}(\mathbf {Z}^T\mathbf {Z})^{-}\mathbf {Z}^T-\mathbf {Z}(\mathbf {Z}^T\mathbf {Z})^-\mathbf {Z}^T \right) \mathbf {X}, \end{aligned}$$

and using the previous result on the limit:

$$\begin{aligned} \lim _{\sigma ^2\rightarrow +\infty }\mathbf {Z}(\mathbf {Z}^T\mathbf {Z})^{-} \left( (\mathbf {Z}^T\mathbf {Z})^{-}+\frac{\mathbf {D}}{\sigma ^2}\right) ^{-1}(\mathbf {Z}^T\mathbf {Z})^{-}\mathbf {Z}^T=\mathbf {Z}(\mathbf {Z}^T\mathbf {Z})^-\mathbf {Z}^T, \end{aligned}$$

it is possible to conclude that the limit reduces to:

A3 $$\begin{aligned} \lim _{\sigma ^2\rightarrow +\infty }\left( \tilde{\mathbf {z}}^T\mathbf {V}_\mathbf {u}\mathbf {Z}^T\mathbf {X}-\tilde{\mathbf {x}}^T\right) \left( \mathbf {X}^T\mathbf {X}\right) ^{-1}\left( \tilde{\mathbf {z}}^T\mathbf {V}_\mathbf {u}\mathbf {Z}^T\mathbf {X}-\tilde{\mathbf {x}}^T\right) ^T. \end{aligned}$$

Hence, solving the deduced quadratic form and computing the limits similarly to (A2), it is finally obtained the result:

$$\begin{aligned} \lim _{\sigma ^2\rightarrow +\infty }\frac{\tilde{\mathbf {q}}^T\mathbf {V}_\beta \tilde{\mathbf {q}}}{\sigma ^2}=\tilde{\mathbf {x}}^T\left( \mathbf {X}^T\mathbf {X}\right) ^{-1}\tilde{\mathbf {x}}. \end{aligned}$$

The concluding algebraic passages are straightforward.

(*ii*) In this case, the integral defining the *r*-th posterior moment of $\theta _m(\tilde{\mathbf {x}})$ might be decomposed as:

$$\begin{aligned} \begin{aligned} \mathbb {E}\left[ \theta _c^r\left( \tilde{\mathbf {x}},\tilde{\mathbf {z}}\right) |\mathbf {w}\right]&=\mathbb {E}\left[ \exp \left\{ r\tilde{\mathbf {x}}^T\varvec{\beta }+\frac{r}{2}\left( \sigma ^2+\sum _{s=1}^q \tau _s^2\right) \right\} \big |\mathbf {w}\right] \\&=\int _{0}^{+\infty }\cdots \int _{0}^{+\infty } g\left( \sigma ^2,\varvec{\tau }^2\right) \exp \left\{ -\frac{1}{2}\left[ \sigma ^2(\gamma _\sigma ^2-r)+\right. \right. \\&\quad \left. \left. +\sum _{s=1}^r\tau _s^2\left( \gamma _{\tau ,s}^2-r\right) - r^2\tilde{\mathbf {x}}^T\mathbf {V}_\beta \tilde{\mathbf {x}} \right] \right\} \mathrm {d}\sigma ^2\mathrm {d}\varvec{\tau }^2. \end{aligned} \end{aligned}$$

In order to check for the finiteness of the previous integral, the term $r^2\tilde{\mathbf {x}}^T\mathbf {V}_\beta \tilde{\mathbf {x}}$ must be checked when all the variance components go to $+\infty$. An upper bound of the integral is:

$$\begin{aligned} \begin{aligned}&\int _{0}^{+\infty }\cdots \int _{0}^{+\infty } g\left( \sigma ^2,\varvec{\tau }^2\right) \exp \left\{ -\frac{1}{2}\left[ \sigma ^2\left( \gamma _\sigma ^2-r- \frac{r^2}{\sigma ^2}\tilde{\mathbf {x}}^T\mathbf {V}_\beta \tilde{\mathbf {x}}\right) +\right. \right. \\&\quad \left. \left. +\sum _{s=1}^r\tau _s^2\left( \gamma _{\tau ,s}^2-r- \frac{r^2}{\tau _s^2}\tilde{\mathbf {x}}^T\mathbf {V}_\beta \tilde{\mathbf {x}}\right) \right] \right\} \mathrm {d}\sigma ^2\mathrm {d}\varvec{\tau }^2. \end{aligned} \end{aligned}$$

The limit for $\sigma ^2\rightarrow +\infty$ gives the same result of point (*i*), whereas the limit for the generic term $\tau ^2_s$ can be written as:

$$\begin{aligned} \begin{aligned} \lim _{\tau _s^2\rightarrow +\infty }&\sigma ^2\tilde{\mathbf {x}}^T\left[ \tau _s^2\mathbf {X}^T\left( \mathbf {I}-\mathbf {P_Z} \right) \mathbf {X}+\right. \\&\quad \left. + \sigma ^2\mathbf {X}^T\left( \mathbf {Z}(\mathbf {Z}^T\mathbf {Z})^{-} \left( \sigma ^2\frac{\left( \mathbf {Z}^T\mathbf {Z}\right) ^{-}}{\tau _s^2} +\frac{\mathbf {D}}{\tau _s^2}\right) ^{-1}(\mathbf {Z}^T\mathbf {Z})^{-}\mathbf {Z}^T\right) \mathbf {X}\right] ^{-1}\tilde{\mathbf {x}} \end{aligned} \end{aligned}$$

By taking the limit $\tau _s^2\rightarrow +\infty$ to the term $\left( \sigma ^2\frac{(\mathbf {Z}^T\mathbf {Z})^{-}}{\tau _s^2}+\frac{\mathbf {D}}{\tau _s^2}\right)$, a matrix $\mathbf {C}_s$ is obtained. All its elements are null with the exception of the presence of $\mathbf {I}_{m_s}$ as block on the diagonal in correspondence to the *s*-th variance component of the random effect and its generalized inverse is the matrix $\mathbf {C}_s$ itself. Therefore, the limit might be written as:

A4 $$\begin{aligned} \lim _{\tau _s^2\rightarrow +\infty }\sigma ^2\tilde{\mathbf {x}}_o^T\left[ \tau _s^2\mathbf {X}_o^T\left( \mathbf {I}-\mathbf {P_Z} \right) \mathbf {X}_o+ \sigma ^2\mathbf {X}_o^T\left( \mathbf {Z}(\mathbf {Z}^T\mathbf {Z})^{-}\mathbf {C}_s (\mathbf {Z}^T\mathbf {Z})^{-}\mathbf {Z}^T\right) \mathbf {X}_o\right] ^{-1}\tilde{\mathbf {x}}_o, \end{aligned}$$

where $\mathbf {X}$ and $\tilde{\mathbf {x}}$ have been replaced, respectively, by $\mathbf {X}_o$ and $\tilde{\mathbf {x}}_o$ without loss of generality. Thanks to this ordered matrix, the first term $\mathbf {A}=\mathbf {X}_o^T\left( \mathbf {I}-\mathbf {P_Z} \right) \mathbf {X}_o$ can be written as:

$$\begin{aligned} \tau ^2_s\mathbf {A}=\left[ \begin{array}{cc} \varvec{0}_{l}&{} \varvec{0}^T\\ \varvec{0}&{} \tau _s^2\mathbf {A}_{2,2} \end{array}\right] , \end{aligned}$$

where $\varvec{0}_{l}$ is the null squared matrix of dimension *l*, that is the rank deficiency of $\mathbf {A}$. This feature is due to the ordering of $\mathbf {X}_o$ and the linear dependence of the first *l* columns of $\mathbf {X}_o$ to the columns of $\mathbf {Z}$. Denoting with $\mathbf {B}_s$ the second matrix, then their sum can be written as:

$$\begin{aligned} \left[ \begin{array}{cc} \mathbf {B}_{s;1,1}&{} \varvec{B}_{s;1,2}^T\\ \varvec{B}_{s;1,2}&{} \tau ^2\mathbf {A}_{2,2}+\mathbf {B}_{s;2,2} \end{array}\right] . \end{aligned}$$

To complete the proof, the result of the limit can be written as:

$$\begin{aligned} \tilde{\mathbf {x}}_o^T\mathbf {L}_s\tilde{\mathbf {x}}_o, \end{aligned}$$

where, exploiting the property of the block matrix:

$$\begin{aligned} \mathbf {L}_s=\left[ \begin{array}{cc} \mathbf {L}_{s;1,1}&{} \varvec{0}\\ \varvec{0}&{} \varvec{0}_{p-l} \end{array}\right] , \end{aligned}$$

and $$\mathbf {L}_{s;1,1}=\mathbf {B}_{s;1,1}^{-1}\in \mathbb {R}^{l\times l}$$.

(*iii*) Recalling the definitions of the posterior predictive distribution (5) and noting that, once defined $\tilde{w}=\log \tilde{y}$, then $\tilde{w}|\varvec{\beta },\mathbf {u}, \sigma ^2\sim \mathcal {N}\left( \tilde{\mathbf {x}}^T\varvec{\beta }+\tilde{\mathbf {z}}^T\mathbf {u},\sigma ^2\right) ,$ the moments of interest might be defined as:

$$\begin{aligned} \mathbb {E}\left[ \tilde{y}^r|\mathbf {y}\right] =\int _{\Theta } \left( \int _{-\infty }^{+\infty } \exp \left\{ r\tilde{w}\right\} p\left( \tilde{w}|\varvec{\beta },\mathbf {u}, \sigma ^2\right) \mathrm {d}\tilde{w}\right) p\left( \mathbf {u},\varvec{\beta }, \sigma ^2,\varvec{\tau }^2|\mathbf {y}\right) \mathrm {d}\varvec{\theta }. \end{aligned}$$

Following algebraic passages similar to the proof of (*i*), the final result is obtained.

## Footnotes

1 For an introduction to Bayesian computation and MCMC methods, see Robert (2007).

2 The expressions (19) and (20) treat variances as known. They only provide a benchmark for comparing the performances of the considered estimators. We expect that their efficiency level can be neared but not reached by estimators that do not assume variances as known.
