# Supplementary material for: Bayesian Analysis of ANOVA and Mixed Models on the Log-Transformed Response Variable
Source: Psychometrika. 2021 Jun 4;86(2):619–41. doi: 10.1007/s11336-021-09769-y (PMC8313477; doi:10.1007/s11336-021-09769-y)
Supplement: Supplementary file 1 — Supplementary material 1 (pdf 845 KB) [file 11336_2021_9769_MOESM1_ESM.pdf]

Supplementary material to: Bayesian analysis of ANOVA and mixed models on the log-transformed response variable

Aldo Gardini* Carlo Trivisano* Enrico Fabrizi †

May 11, 2021

This document contains the supplementary material to the paper *Bayesian analysis of ANOVA and mixed models on the log-transformed response variable* and it is organized as follows. In section S1 we complement the discussion on the choice of prior specification for the hyper-parameters γ contained in section 4.1 of the main paper. In section S2, the minimum MSE estimator conditioned to the variance components of the overall mean θ_m is derived and its connection to the Bayesian framework is explained. This quantity is used as benchmark in the simulation study. In Section S3, some additional tables concerning the results of the simulation discussed in Section 5 of the paper are reported. Section S4 contains an additional simulation study in which covariates are included in the model and the frequentist properties of the posterior predictive distribution are investigated. Section S5 reports the information about the convergence diagnostics of the MCMC algorithm used to fit the models compared in the application of Section 6. Eventually, the proof of Corollary 1 and some software details useful to estimate models with dependent random effects are contained in Section S6.

S1 A simulation exercise for the choice of γ

The conditions on the existence of moments in theorem 1 are expressed as lower bounds for γ parameters. In section 4.1, we suggest for the γs to set a priori values close, but somewhat larger, than the lower bounds stated in the theorem. The reason is that a value of γ very close to the lower bound would cause numerical instability in the estimation of moments from the relevant posteriors (technically the integral is finite, but very large). Specifically, provided r is the moment order in whose finiteness we are interested, we suggest to set γ to the lower bound that warrants the existence of the $r + c$ moment where c is a *small* constant. The aim of this simulation exercise is to show why setting $c > 0$ is necessary and to justify that $c \geq 0.5$ is a sensible choice. To stay safe on the numerical stability side, in the simulations and applications of the main paper we set $c = 1$.

To the purposes of this section, we consider a single scenario from the simulation study described in section 5 (specifically: $n_j = 2$, $\sigma^2 = 0.5$, $\phi = 1$). We focus on the functional θ_m. The γ_m parameter of prior (16) is fixed as to guarantee the existence of the $r + c$ moment with $r = 2$. Three different choices of c, i.e. $c = 0.01, 0.5, 1$ are compared. Figure S1 reports the Monte Carlo distributions (based on $B = 10000$ replicates) of posterior standard deviations $sd(\theta_m|\mathbf{w})$. The numerical instability associated to very small c, i.e. $c = 0.01$, is apparent from the heavy tail of

*Dipartimento di Scienze Statistiche 'P. Fortunati', Università di Bologna

†DISES, Università Cattolica del S. Cuore

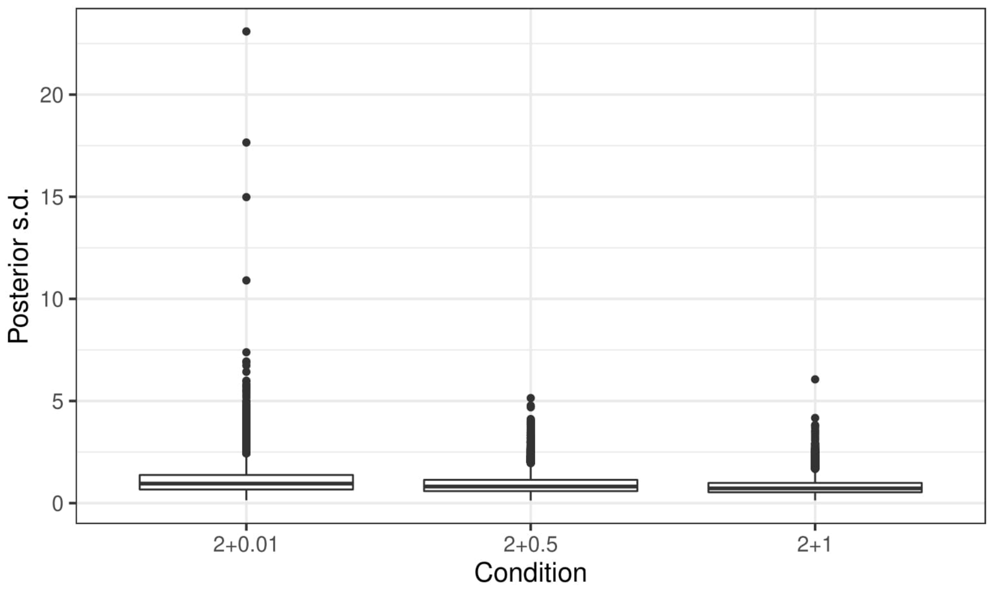

Figure S1: Monte Carlo distributions of the posterior standard deviation of θ_m under different choices of c.

the MC distribution. When c increases and γ_m is well above the lower bound for the existence of second moment, large outliers (caused by numerical instability) disappear.

S2 Minimum MSE estimator conditioned to the variance components

In order to have a complete characterization of the estimation problem, a useful finding might be the minimum MSE Bayes estimator, conditioned with respect to the variance components. It is the parallel result of the one by Zellner (1971) for the log-normal mean. Even if the deduced estimator could be of little practical interest, it might represent an useful benchmark for the considered methods in the simulation study.

For computational easiness, the one-way random effect model (9) in the balanced case (i.e. $n_j = n_g,\ \forall j$) is considered. Moreover, we include in the model only a general mean term $\mathbf{x}_{ij}^T\boldsymbol{\beta} = \mu,\ \forall i, j$. Assuming the variance components σ^2 and τ^2 as known, the only unknown parameter is the global mean in the log-scale μ. Similarly to Zellner (1971), the research of an optimal conditional estimator is restricted to the class of estimators $\theta_m^* = \exp\{\bar{w}\}\, k$. The main result and its relationship with the Bayesian estimation is contained in the following theorem.

Proposition 1. *Considering the estimators of the functional* $\theta_m = \exp\{\mu + 2^{-1}(\sigma^2 + \tau^2)\}$ *that consider* σ^2 *and* τ^2 *as known and are included in the class:*

$$\theta_m^* = k \cdot \exp\{\bar{w}\};$$

then the one that minimizes the frequentist MSE is:

$$\hat{\theta}_m^* = \exp\left\{\bar{w} + \frac{\sigma^2 + \tau^2}{2} - \frac{3(\sigma^2 + n_g\tau^2)}{2n}\right\}.$$

Furthermore, it coincides with the conditioned Bayes estimator under the prior $p(\mu) \propto 1$ that minimizes the relative quadratic loss function.

Proof. Recalling that $\bar{w} \sim \mathcal{N}\left(\mu, \sigma^2 n^{-1} + \tau^2 m^{-1}\right)$, the MSE of the considered class of estimator is:

$$\mathbb{E}[(\theta_m^* - \theta_m)^2] = k^2 \exp\left\{2\left(\mu + \sigma^2 n^{-1} + \tau^2 m{-}1\right)\right\} + \\ - 2k \exp\left\{2\mu + \frac{\sigma^2 m + \tau^2 n + nm(\sigma^2 + \tau^2)}{2nm}\right\} + c,$$

where c is a constant. The quantity is minimized when:

$$k = \exp\left\{\frac{\sigma^2 + \tau^2}{2} - \frac{3(\sigma^2 + n_g \tau^2)}{2n}\right\}.$$

Starting from the fact that:

$$\theta_m | \sigma^2, \tau^2, \mathbf{w} \sim \log\mathcal{N}\left(\bar{\mu} + \frac{\sigma^2 + \tau^2}{2}, \frac{\sigma^2 + n_g \tau^2}{n}\right),$$

the expression of the Bayes estimator under relative quadratic loss can be easily derived. □

Even if it is not proved to be an optimal estimator, its use for benchmarking purposes appears to be largely justified by the good frequentist properties that the Bayes estimator under relative quadratic loss has in the log-normal estimation framework. The formal result is presented in the following proposition.

Proposition 2. *The Bayes estimator of $\theta_c(v_j)$ conditioned with respect to the variance components under the prior $p(\mu) \propto 1$ that minimizes the relative quadratic loss function is:*

$$\hat{\theta}_c^{RQ}(v_l) = \exp\left\{\frac{\sigma^2}{\sigma^2 + n_g \tau^2}\left(\frac{\tau^2 n_g}{\sigma^2}\bar{w}_{.j} - \bar{w}\right) + \frac{\sigma^2}{2} - \frac{3}{2}\frac{\sigma^2}{\sigma^2 + n_g \tau^2}\left(\tau^2 + \frac{\sigma^2}{n}\right)\right\}.$$

Proof. To obtain the estimator, the distribution of $\theta_c(v_l)|\sigma^2, \tau^2, \mathbf{w}$ must be deduced removing the conditioning on $\boldsymbol{v}$ from the marginal posterior:

$$\theta_c(v_j)|\boldsymbol{v}, \sigma^2, \tau^2, \mathbf{w} \sim \log\mathcal{N}\left(\frac{\sum_{l=1}^m (\bar{w}_{.l} - v_l)}{m} + v_j + \frac{\sigma^2}{2}, \frac{\sigma^2}{n}\right).$$

Setting the value $t_j = \mu + v_j$, the following result can be obtained

$$t_j | \sigma^2, \tau^2, \mathbf{w} \sim \mathcal{N}\left(\bar{t}_j, V_{t_j}\right),$$

where:

$$\bar{t}_j = \frac{\frac{n_g}{\sigma^2}\bar{w}_{.l}}{\frac{1}{\tau^2} + \frac{n_g}{\sigma^2}} - \frac{\sigma^2}{\sigma^2 + n_g \tau^2}\bar{w}, \qquad V_{t_j} = \frac{\sigma^2}{\sigma^2 + n_g \tau^2}\left(\tau^2 + \frac{\sigma^2}{n}\right).$$

Recalling that the Bayes estimator under relative quadratic loss in a log-normal context is:

$$\hat{\theta}_c^{cB}(v_j) = \exp\left\{\bar{t}_j + \frac{\sigma^2}{2} - \frac{3}{2}V_{t_j}\right\},$$

the final result is obtained by substitution. □

Table S1: Bias and RMSE for the considered estimators of θ_m in the different scenarios with $n_g = 5$.

			θ_m^c		θ_m^{IG}		θ_m^J		θ_m^{GIG}	
ϕ	σ^2	θ_m	Bias	RMSE	Bias	RMSE	Bias	RMSE	Bias	RMSE
0.5	0.05	1.038	-0.004	0.062	0.187	0.202	0.008	0.064	0.019	0.068
	0.25	1.206	-0.023	0.159	0.267	0.342	0.056	0.199	0.085	0.208
	0.5	1.455	-0.053	0.271	0.435	1.082	6.181	255.860	0.157	0.385
	0.75	1.755	-0.094	0.398	46.354	$> 10^4$	$> 10^4$	$> 10^4$	0.220	0.588
1	0.05	1.051	-0.007	0.082	0.192	0.217	0.016	0.089	0.030	0.094
	0.25	1.284	-0.039	0.224	0.308	0.444	0.151	0.767	0.123	0.306
	0.5	1.649	-0.098	0.403	0.743	3.341	$> 10^4$	$> 10^4$	0.212	0.588
	0.75	2.117	-0.185	0.628	$> 10^4$	$> 10^4$	$> 10^4$	$> 10^4$	0.270	0.928
2	0.05	1.078	-0.012	0.115	0.202	0.248	0.032	0.131	0.050	0.139
	0.25	1.455	-0.079	0.343	25.052	$> 10^4$	$> 10^4$	$> 10^4$	0.178	0.490
	0.5	2.117	-0.221	0.696	$> 10^4$	$> 10^4$	$> 10^4$	$> 10^4$	0.251	1.014
	0.75	3.08	-0.467	1.223	$> 10^4$	$> 10^4$	$> 10^4$	$> 10^4$	0.205	1.744

S3 Additional tables on simulations

The tables reported in this section refer to the simulation exercise of section 5 of the paper. In the first place, tables S1 and S2 contain the results for posterior means of θ_m and $\theta_c(v_j)$ for the scenarios characterized by $n_j = 5$, thereby complementing tables 1 and 2. Results concerning the frequentist properties of the credible intervals are showed in table S3 for the overall mean predictor and S4 for the group means.

Eventually, the results concerning three further prior specifications with respect to those mentioned in the simulation design of section 5 are reported in tables S5 - S8. Specifically, the following estimators are considered:

a-b) the posterior means of θ_m and $\theta_c(v_j)$ when priors are:

$$p(\mu) \propto 1, \;\; \sigma^2 \sim GIG(1, 0.001, \gamma_{\mathrm{m}}), \; \tau^2 \sim GIG(1, 0.001, \gamma_{\mathrm{m}}),$$

and:

$$p(\mu) \propto 1, \;\; \sigma^2 \sim GIG(1, 0.1, \gamma_{\mathrm{m}}), \; \tau^2 \sim GIG(1, 0.1, \gamma_{\mathrm{m}}),$$

where $\gamma_{\mathrm{m}} = \max\{\gamma_\sigma, \gamma_{\tau,1}\} = \sqrt{3 + 3^2 m^{-1}}$. The predictors will be denoted as $\hat{\theta}_m^{GIG_{0.001}}$, $\hat{\theta}_c^{GIG_{0.001}}(v_j)$ for the first prior setting and $\hat{\theta}_m^{GIG_{0.1}}$, $\hat{\theta}_c^{GIG_{0.1}}(v_j)$ for the second one. The aim of these additional simulations is to assess the prior sensitivity of posterior distributions with respect to alternative choices of the GIG scale parameter δ.

c) the posterior means of θ_m and $\theta_c(v_j)$ when priors are:

$$p(\mu) \propto 1, \;\; \sigma^2 \sim IG(1, 0.001), \;\; \tau^2 \sim IG(1, 0.001),$$

that will be labelled as $\hat{\theta}_m^{IG_2}$ and $\hat{\theta}_c^{IG_2}(v_j)$. This latter set of prior specifications complements the comparison with the family of inverse-gamma priors for the variance parameters, as this specification is very popular in applications.

Table S2: RABias and RRMSE for the considered estimators of the group-specific expectations in the different scenarios with $n_g = 5$.

ϕ	σ^2	$\theta_c^c(v_j)$		$\theta_c^{IG}(v_j)$		$\theta_c^J(v_j)$		$\theta_c^{GIG}(v_j)$	
		RABias	RRMSE	RABias	RRMSE	RABias	RRMSE	RABias	RRMSE
0.5	0.05	0.008	0.087	0.038	0.104	0.009	0.092	0.010	0.090
	0.25	0.037	0.192	0.069	0.230	0.050	0.228	0.049	0.218
	0.5	0.073	0.268	0.111	0.342	0.109	0.362	0.095	0.334
	0.75	0.106	0.325	0.157	0.447	0.175	0.496	0.138	0.438
1	0.05	0.009	0.092	0.038	0.106	0.010	0.098	0.011	0.096
	0.25	0.043	0.204	0.073	0.240	0.056	0.241	0.056	0.234
	0.5	0.083	0.285	0.123	0.366	0.119	0.382	0.109	0.363
	0.75	0.121	0.345	0.178	0.488	0.191	0.527	0.160	0.482
2	0.05	0.010	0.096	0.038	0.107	0.010	0.100	0.012	0.099
	0.25	0.046	0.212	0.077	0.249	0.057	0.245	0.059	0.243
	0.5	0.090	0.295	0.132	0.385	0.122	0.388	0.117	0.380
	0.75	0.131	0.357	0.194	0.519	0.195	0.533	0.173	0.509

S4 Simulation study: evaluations of effects and predictions

In this section we carry out an additional simulation study in which the data generating process is characterized by the presence of a continuous covariate. We have two main aims: exploring if different priors on the variance components change the frequentist properties of the regression coefficients' estimates, and evaluating the performances of the posterior predictive distributions. The following special case of model (9) is considered:

$$\log(y_{ij}) = \beta_0 + x_{1,ij}\beta_1 + v_j + \varepsilon_{ij};\ j = 1, ..., m;\ i = 1, ..., n_j;$$

where $m = 10$ and $n_j = 2, 5$. In line with the simulation study in Fabrizi and Trivisano (2016), the covariate is fixed and generated once from a uniform distribution: $x_{ij} \overset{ind}{\sim} \mathcal{U}(0, 1)$, and $(\beta_0, \beta_1) = (1, 1)$. As far as the variance components are concerned, two different scenarios are considered: $(\sigma^2, \tau^2) \in \{(0.15, 0.1), (0.3, 0.2)\}$, inducing overall variances in the log-scale equal to 0.25 and 0.5. To sum up, 4 scenarios are taken into consideration. Since we focus also on the posterior predictive distribution, a grid of covariate values needs to be specified for prediction. We define them as $\tilde{\mathbf{x}}_k = c(1, \tilde{x}_{1,k})$, where $\tilde{x}_{1,k} = 0, 0.1, \ldots, 1.1, 1.2$ following Fabrizi and Trivisano (2016).

As in section 5, the results obtained under the following priors for the variance components are compared: $GIG(1, 0.01, \gamma_m)$, $IG(1, 1)$, and $IG(0.001, 0.001)$, where γ_m is fixed to fulfill the condition *iii)* of theorem 1, computing the leverage for each vector $\tilde{\mathbf{x}}_k$. Diffuse priors on the regression coefficients are set to complete the model specification.

The estimates of β_1 are compared in terms of bias, RMSE, frequentist coverage, and average posterior intervals width in tables S9 and S10. The results about the regression coefficient exhibit minor changes from one prior specification to another, whereas some problems emerge for the intervals under the $IG(1, 1)$ prior: they are significantly larger than those obtained with the other priors, affected by over-coverage especially when $n_j = 2$.

Eventually, in figure S2 results (bias, log RMSE, frequentist coverage, and average width) concerning the predictions at each covariate values $\tilde{x}_{1,k}$ are reported for the different scenarios. Focusing

Table S3: Frequentist coverage and width for the credible intervals of θ_m in the different scenarios.

			θ_m^c		θ_m^{IG}		θ_m^J		θ_m^{GIG}	
n_j	ϕ	σ^2	Cov	Width	Cov	Width	Cov	Width	Cov	Width
2	0.5	0.05	0.949	0.289	0.994	1.294	0.953	0.340	0.977	0.433
		0.25	0.949	0.769	0.954	2.174	0.941	1.090	0.961	1.237
		0.5	0.949	1.349	0.950	3.762	0.939	2.539	0.954	2.190
		0.75	0.949	2.050	0.952	6.258	0.939	5.273	0.954	3.238
	1	0.05	0.953	0.360	0.979	1.347	0.947	0.423	0.968	0.528
		0.25	0.953	1.017	0.947	2.611	0.933	1.627	0.947	1.578
		0.5	0.953	1.927	0.947	5.354	0.931	4.922	0.945	2.909
		0.75	0.953	3.162	0.952	10.695	0.929	14.240	0.946	4.454
	2	0.05	0.953	0.479	0.966	1.467	0.935	0.595	0.958	0.699
		0.25	0.953	1.532	0.944	3.817	0.932	3.420	0.942	2.259
		0.5	0.953	3.384	0.948	11.722	0.930	19.592	0.931	4.466
		0.75	0.953	6.475	0.952	39.113	0.932	139.616	0.907	7.275
5	0.5	0.05	0.951	0.242	0.998	0.937	0.949	0.272	0.971	0.334
		0.25	0.951	0.637	0.976	1.515	0.938	0.820	0.962	0.931
		0.5	0.951	1.108	0.965	2.475	0.933	1.738	0.955	1.647
		0.75	0.951	1.669	0.963	3.833	0.936	3.242	0.954	2.466
	1	0.05	0.947	0.321	0.991	0.997	0.939	0.375	0.964	0.447
		0.25	0.947	0.901	0.966	1.929	0.936	1.368	0.953	1.312
		0.5	0.947	1.693	0.957	3.779	0.933	3.751	0.945	2.422
		0.75	0.948	2.752	0.956	7.148	0.933	9.488	0.941	3.739
	2	0.05	0.946	0.448	0.979	1.121	0.938	0.560	0.955	0.635
		0.25	0.946	1.423	0.959	3.066	0.938	3.008	0.944	2.024
		0.5	0.946	3.116	0.954	9.244	0.941	15.489	0.932	4.009
		0.75	0.946	5.911	0.952	29.254	0.940	95.540	0.908	6.648

Table S4: Average frequentist coverage and average width for the credible intervals of group means $\theta_c(v_j)$.

			$\theta_c^c(v_j)$		$\theta_c^{IG}(v_j)$		$\theta_c^J(v_j)$		$\theta_c^{GIG}(v_j)$	
n_j	ϕ	σ^2	ACov	AWidth	ACov	AWidth	ACov	AWidth	ACov	AWidth
2	0.5	0.05	0.947	0.466	1.000	1.324	0.931	0.488	0.964	0.575
		0.25	0.923	1.104	0.988	2.145	0.917	1.335	0.956	1.526
		0.5	0.894	1.677	0.975	3.426	0.915	2.557	0.949	2.617
		0.75	0.868	2.205	0.967	5.160	0.912	4.378	0.942	3.821
	1	0.05	0.949	0.533	1.000	1.355	0.927	0.564	0.962	0.642
		0.25	0.930	1.325	0.985	2.364	0.917	1.669	0.951	1.787
		0.5	0.908	2.139	0.969	4.088	0.917	3.501	0.940	3.235
		0.75	0.885	2.989	0.959	6.658	0.915	6.486	0.932	4.976
	2	0.05	0.949	0.591	0.999	1.417	0.935	0.662	0.962	0.728
		0.25	0.936	1.626	0.980	2.823	0.930	2.218	0.950	2.226
		0.5	0.917	2.977	0.965	5.642	0.930	5.370	0.940	4.486
		0.75	0.897	4.718	0.955	10.643	0.928	12.108	0.930	7.633
5	0.5	0.05	0.938	0.342	0.993	0.574	0.930	0.352	0.950	0.376
		0.25	0.898	0.806	0.967	1.150	0.923	0.930	0.946	0.991
		0.5	0.846	1.216	0.959	1.904	0.921	1.650	0.943	1.709
		0.75	0.795	1.588	0.953	2.830	0.920	2.547	0.938	2.533
	1	0.05	0.939	0.371	0.992	0.584	0.938	0.387	0.951	0.403
		0.25	0.905	0.917	0.964	1.239	0.935	1.088	0.947	1.116
		0.5	0.860	1.471	0.954	2.199	0.935	2.060	0.941	2.041
		0.75	0.815	2.043	0.949	3.492	0.934	3.390	0.937	3.200
	2	0.05	0.941	0.395	0.992	0.601	0.945	0.416	0.952	0.428
		0.25	0.908	1.079	0.963	1.424	0.944	1.294	0.947	1.307
		0.5	0.868	1.962	0.952	2.876	0.942	2.785	0.943	2.689
		0.75	0.826	3.089	0.947	5.210	0.942	5.211	0.938	4.743

Table S5: Bias and RMSE for the considered estimators of θ_m in the different scenarios.

				$\theta_m^{IG_2}$		$\theta_m^{GIG_{0.001}}$		$\theta_m^{GIG_{0.1}}$	
n_j	ϕ	σ^2	θ_m	Bias	RMSE	Bias	RMSE	Bias	RMSE
2	0.5	0.05	1.038	0.004	0.075	0.034	0.086	0.038	0.088
		0.25	1.206	0.029	0.219	0.139	0.281	0.144	0.284
		0.5	1.455	0.090	0.439	0.240	0.516	0.246	0.520
		0.75	1.755	0.274	1.947	0.317	0.775	0.324	0.778
	1	0.05	1.051	0.006	0.095	0.044	0.111	0.047	0.113
		0.25	1.284	0.048	0.305	0.172	0.373	0.176	0.375
		0.5	1.649	0.189	0.795	0.283	0.714	0.287	0.713
		0.75	2.117	499.684	$> 10^4$	0.348	1.114	0.351	1.111
	2	0.05	1.078	0.010	0.130	0.063	0.154	0.066	0.156
		0.25	1.455	0.136	0.667	0.223	0.557	0.226	0.557
		0.5	2.117	$> 10^4$	$> 10^4$	0.314	1.151	0.316	1.145
		0.75	3.08	$> 10^4$	$> 10^4$	0.251	1.951	0.251	1.941
5	0.5	0.05	1.038	0.001	0.063	0.019	0.068	0.021	0.069
		0.25	1.206	0.010	0.174	0.084	0.208	0.087	0.210
		0.5	1.455	0.036	0.333	0.155	0.385	0.159	0.388
		0.75	1.755	0.092	0.570	0.217	0.588	0.223	0.593
	1	0.05	1.051	0.003	0.085	0.030	0.094	0.031	0.095
		0.25	1.284	0.031	0.263	0.121	0.305	0.124	0.308
		0.5	1.649	0.140	0.704	0.209	0.585	0.214	0.592
		0.75	2.117	8.137	299.176	0.268	0.928	0.275	0.936
	2	0.05	1.078	0.010	0.122	0.049	0.139	0.051	0.140
		0.25	1.455	0.134	0.752	0.175	0.489	0.179	0.494
		0.5	2.117	436.861	$> 10^4$	0.248	1.012	0.255	1.022
		0.75	3.08	$> 10^4$	$> 10^4$	0.197	1.734	0.205	1.748

Table S6: Frequentist coverage and width for the credible intervals of θ_m in the different scenarios.

			$\theta_m^{IG_2}$		$\theta_m^{GIG_{0.001}}$		$\theta_m^{GIG_{0.1}}$	
n_j	ϕ	σ^2	Cov	Width	Cov	Width	Cov	Width
2	0.5	0.05	0.272	0.913	0.975	0.432	0.983	0.451
		0.25	0.783	0.901	0.958	1.236	0.962	1.252
		0.5	1.577	0.897	0.951	2.185	0.955	2.206
		0.75	2.815	0.904	0.953	3.233	0.956	3.256
	1	0.05	0.331	0.895	0.968	0.526	0.973	0.540
		0.25	1.051	0.884	0.948	1.573	0.955	1.585
		0.5	2.430	0.878	0.946	2.900	0.949	2.910
		0.75	5.073	0.885	0.942	4.448	0.947	4.451
	2	0.05	0.455	0.882	0.962	0.695	0.965	0.706
		0.25	1.848	0.875	0.946	2.248	0.947	2.255
		0.5	6.247	0.875	0.933	4.456	0.933	4.463
		0.75	22.913	0.866	0.903	7.258	0.906	7.270
5	0.5	0.05	0.219	0.896	0.967	0.333	0.975	0.347
		0.25	0.591	0.875	0.959	0.927	0.962	0.939
		0.5	1.114	0.874	0.955	1.641	0.954	1.653
		0.75	1.852	0.871	0.951	2.456	0.954	2.475
	1	0.05	0.306	0.887	0.966	0.446	0.965	0.455
		0.25	0.961	0.880	0.950	1.304	0.951	1.315
		0.5	2.176	0.877	0.944	2.409	0.948	2.428
		0.75	4.379	0.870	0.940	3.736	0.944	3.758
	2	0.05	0.457	0.904	0.955	0.631	0.958	0.640
		0.25	1.892	0.899	0.946	2.008	0.944	2.025
		0.5	6.323	0.894	0.932	3.995	0.933	4.020
		0.75	20.936	0.891	0.907	6.609	0.908	6.643

Table S7: RABias and RRMSE for the considered estimators of the group-specific expectations in the different scenario.

			$\theta_c^{IG_2}(v_j)$		$\theta_c^{GIG_{0.001}}(v_j)$		$\theta_c^{GIG_{0.1}}(v_j)$	
n_j	ϕ	σ^2	RABias	RRMSE	RABias	RRMSE	RABias	RRMSE
2	0.5	0.05	0.019	0.140	0.024	0.128	0.025	0.128
		0.25	0.127	0.415	0.109	0.330	0.109	0.329
		0.5	0.300	0.775	0.198	0.529	0.197	0.527
		0.75	0.531	1.282	0.273	0.713	0.272	0.710
	1	0.05	0.032	0.181	0.030	0.145	0.031	0.144
		0.25	0.234	0.641	0.143	0.394	0.143	0.392
		0.5	0.611	1.454	0.272	0.667	0.270	0.663
		0.75	1.198	2.920	0.393	0.950	0.391	0.943
	2	0.05	0.046	0.217	0.037	0.160	0.038	0.159
		0.25	0.401	1.034	0.183	0.464	0.182	0.461
		0.5	1.278	3.468	0.364	0.842	0.362	0.837
		0.75	3.532	14.667	0.549	1.282	0.546	1.272
5	0.5	0.05	0.009	0.101	0.010	0.090	0.010	0.090
		0.25	0.064	0.281	0.049	0.218	0.049	0.217
		0.5	0.153	0.487	0.095	0.333	0.095	0.332
		0.75	0.258	0.719	0.137	0.438	0.137	0.436
	1	0.05	0.010	0.105	0.011	0.096	0.011	0.096
		0.25	0.066	0.285	0.055	0.234	0.055	0.233
		0.5	0.154	0.500	0.109	0.363	0.109	0.362
		0.75	0.267	0.772	0.160	0.482	0.160	0.481
	2	0.05	0.010	0.103	0.012	0.099	0.012	0.099
		0.25	0.057	0.263	0.059	0.243	0.059	0.243
		0.5	0.127	0.443	0.117	0.380	0.117	0.380
		0.75	0.208	0.646	0.174	0.510	0.174	0.509

Table S8: Average frequentist coverage and average width for the credible intervals of group means $\theta_c(v_j)$ in the different scenarios.

			$\theta_c^{IG_2}(v_j)$		$\theta_c^{GIG_{0.001}}(v_j)$		$\theta_c^{GIG_{0.1}}(v_j)$	
n_j	ϕ	σ^2	ACov	AWidth	ACov	AWidth	ACov	AWidth
2	0.5	0.05	0.829	0.383	0.963	0.575	0.969	0.593
		0.25	0.723	0.924	0.956	1.526	0.957	1.542
		0.5	0.692	1.709	0.948	2.616	0.949	2.633
		0.75	0.679	2.920	0.940	3.820	0.943	3.840
	1	0.05	0.821	0.464	0.961	0.641	0.965	0.653
		0.25	0.711	1.230	0.950	1.787	0.951	1.796
		0.5	0.671	2.529	0.941	3.235	0.941	3.243
		0.75	0.655	4.807	0.931	4.968	0.933	4.980
	2	0.05	0.853	0.587	0.962	0.728	0.965	0.735
		0.25	0.762	1.859	0.950	2.227	0.951	2.233
		0.5	0.716	4.620	0.939	4.483	0.940	4.488
		0.75	0.693	12.920	0.930	7.624	0.930	7.631
5	0.5	0.05	0.876	0.317	0.950	0.376	0.955	0.382
		0.25	0.808	0.777	0.947	0.991	0.949	0.996
		0.5	0.772	1.321	0.943	1.709	0.944	1.715
		0.75	0.752	1.991	0.939	2.532	0.940	2.539
	1	0.05	0.908	0.367	0.951	0.403	0.953	0.405
		0.25	0.880	1.001	0.947	1.116	0.947	1.118
		0.5	0.863	1.850	0.942	2.039	0.942	2.043
		0.75	0.851	2.990	0.937	3.200	0.937	3.205
	2	0.05	0.931	0.402	0.953	0.428	0.954	0.430
		0.25	0.922	1.231	0.947	1.307	0.948	1.308
		0.5	0.918	2.598	0.943	2.691	0.943	2.693
		0.75	0.916	4.786	0.939	4.739	0.938	4.744

Scenario	σ^2	τ^2	n_j	$IG(0.001, 0.001)$ Bias	RMSE	$IG(1, 1)$ Bias	RMSE	$GIG(1, 0.01, \gamma_m)$ Bias	RMSE
1	0.15	0.1	2	0.000	0.304	0.000	0.304	0.001	0.302
2	0.30	0.2	2	0.002	0.432	0.001	0.429	0.001	0.428
3	0.15	0.1	5	-0.005	0.229	-0.003	0.229	-0.005	0.228
4	0.30	0.2	5	-0.008	0.324	-0.005	0.323	-0.007	0.322

Table S9: Bias and RMSE of the posterior mean of β_1 under three different priors for the variance components.

Scenario	σ^2	τ^2	n_j	$IG(0.001, 0.001)$ Width	Cov	$IG(1, 1)$ Width	Cov	$GIG(1, 0.01, \gamma_m)$ Width	Cov
1	0.15	0.1	2	1.332	0.959	1.771	0.997	1.277	0.955
2	0.30	0.2	2	1.891	0.960	2.114	0.982	1.697	0.942
3	0.15	0.1	5	0.888	0.940	1.011	0.971	0.887	0.944
4	0.30	0.2	5	1.256	0.941	1.328	0.958	1.229	0.938

Table S10: Frequentist coverage and average width of the 95% credible intervals of β_1 under three different priors for the variance components.

on the logarithm of the RMSE, numerical evidences of the moment indefiniteness can be noted for the inverse gamma priors as occasional peaks, likely due to numerical extreme associated to non-existing moments, appear.

S5 Model convergence

All the models related to simulations and the application in section 6 were fitted using MCMC algorithms. With specific reference to those of the application a total of 105000 iterations were produced, the first 5000 were discarded as burn-in (or warm-up for `Stan` models, Carpenter et al., 2017), and the remaining 100000 iterations were thinned by 10. In the first place, the convergence of the basic model parameters were monitored by visual inspection of chains. In all cases we have satisfying chains, both in terms of stationarity and quality of mixing. As an example, in figure S3, the traceplots, histograms, and autocorrelation function are reported for the model fitted on the reduced data with GIG priors for the variance components.

S6 Model with dependent random effects

S6.1 Proof of Corollary 1

To adapt the proof of Theorem 1 to the model considered in this corollary, it is useful to consider the decomposition $\mathbf{D} = \mathbf{L}\boldsymbol{\Lambda}_{\mathbf{D}}\mathbf{L}^T$, where $\boldsymbol{\Lambda}_{\mathbf{D}}$ is a diagonal matrix containing the variance components (in this case τ_0^2 and τ_1^2) and $\mathbf{L}$ is the Cholesky factor of the correlation matrix $\mathbf{R}$ ($\mathbf{D} = \boldsymbol{\Lambda}_{\mathbf{D}}^{\frac{1}{2}}\mathbf{R}\boldsymbol{\Lambda}_{\mathbf{D}}^{\frac{1}{2}}$). The proofs concerning conditions *i)* and *iii)* are based on result (A1), that requires a diagonal matrix for being effective. Exploiting the previous decomposition of $\mathbf{D}$, it is possible to show that

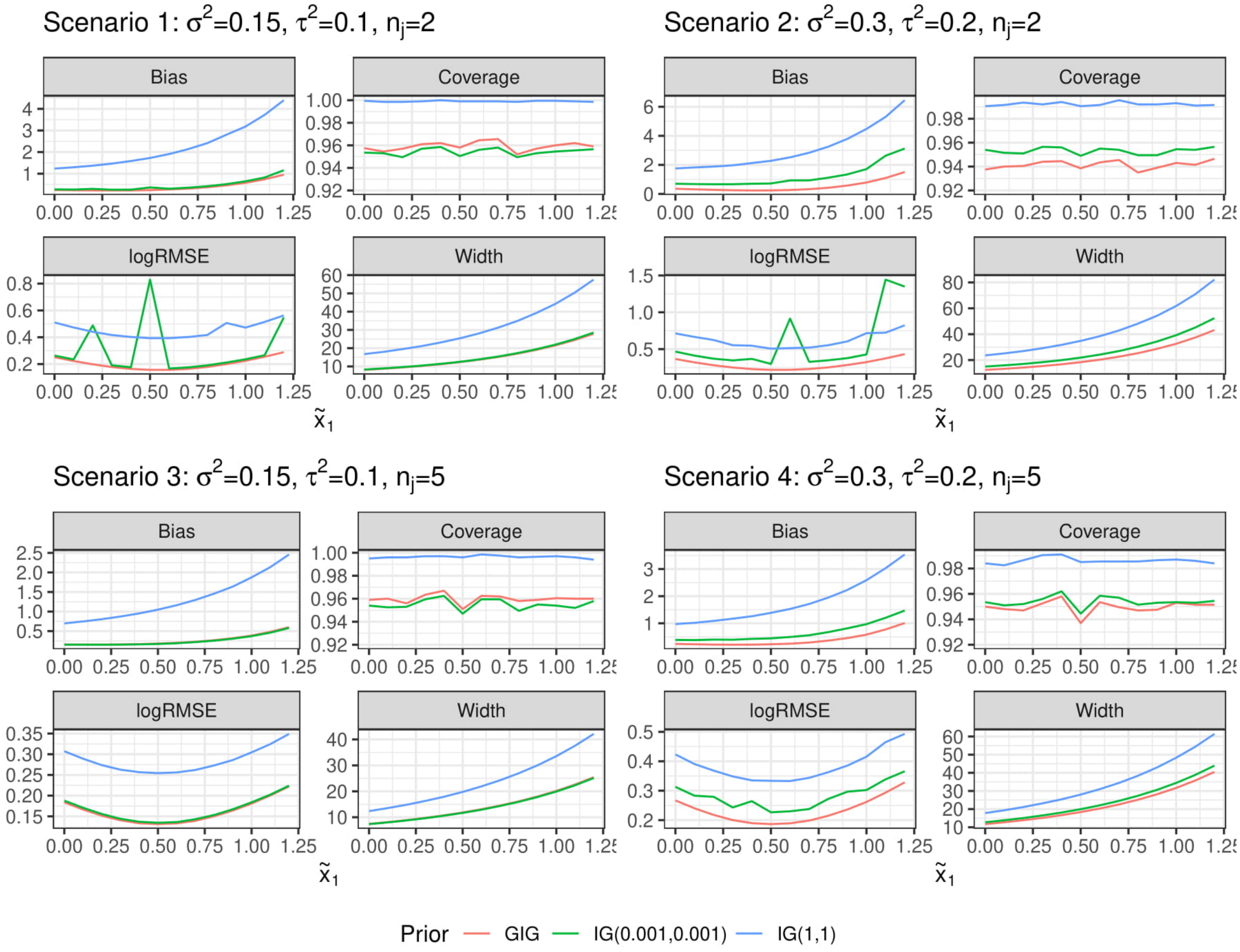

Figure S2: Bias, log RMSE, frequentist coverage, and average width of the predictions at different covariate values $\tilde{x}_{1,k}$.

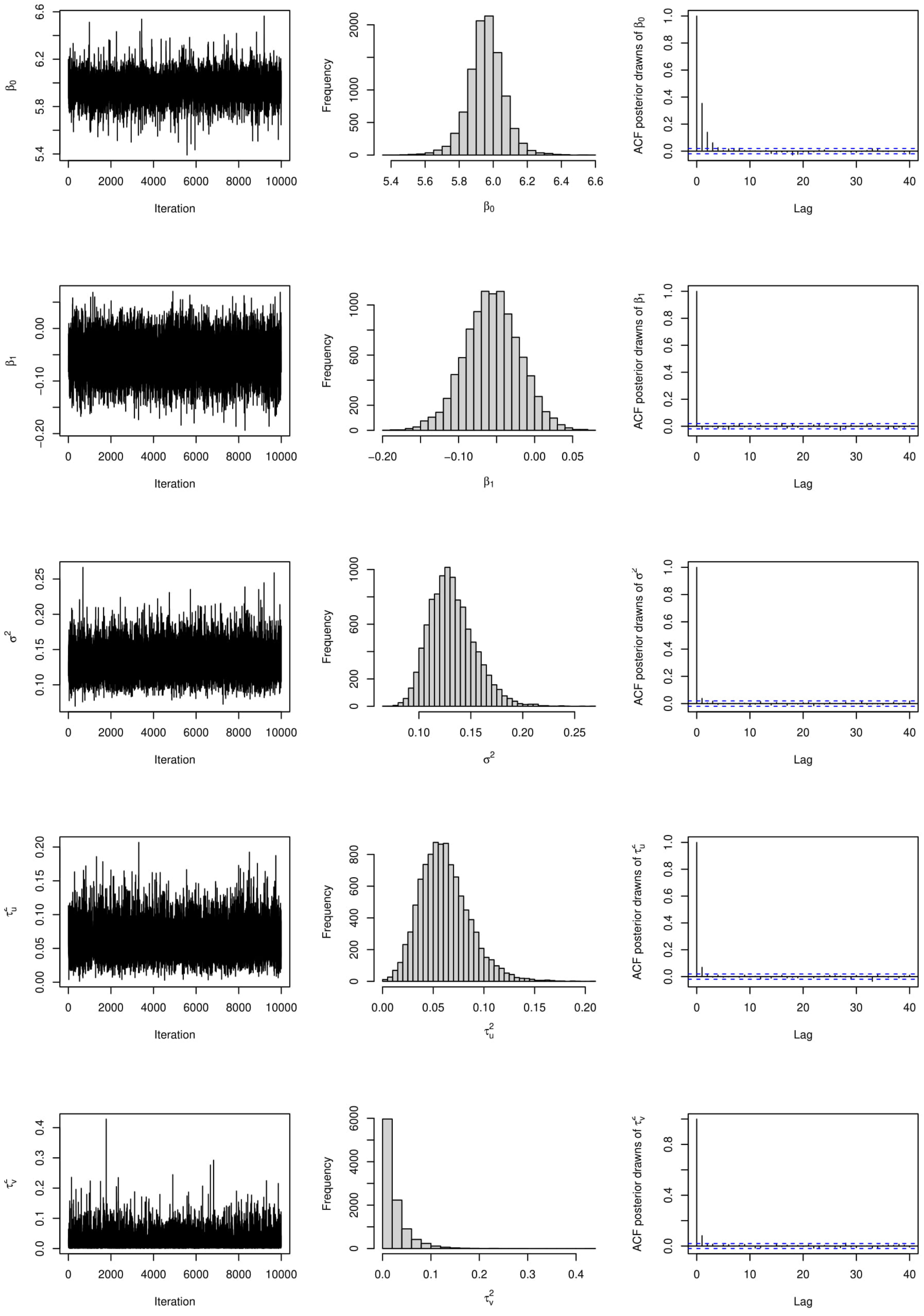

Figure S3: Plots concerning the convergence of the MCMC algorithm for the basic parameters the model fitted under GIG priors on the reduced dataset.

the result (A3) still holds since the (A1) can be used after some algebra:

$$\begin{aligned}\lim_{\sigma^2\to+\infty}\left((\mathbf{Z}^T\mathbf{Z})^- + \frac{\mathbf{D}}{\sigma^2}\right)^{-1} &= \lim_{\sigma^2\to+\infty}\left((\mathbf{Z}^T\mathbf{Z})^- + \frac{\mathbf{L}\boldsymbol{\Lambda}_{\mathbf{D}}\mathbf{L}^T}{\sigma^2}\right)^{-1} \\ &= \lim_{\sigma^2\to+\infty}\left(\mathbf{L}^T\right)^{-1}\left(\mathbf{L}^T\mathbf{R}^{-1}(\mathbf{Z}^T\mathbf{Z})^-\mathbf{R}^{-1}\mathbf{L} + \frac{\boldsymbol{\Lambda}_{\mathbf{D}}}{\sigma^2}\right)^{-1}\mathbf{L}^{-1} = \mathbf{Z}^T\mathbf{Z}.\end{aligned}$$

Focusing on condition *ii)*, the result in (A4) can also be retrieved with the considered structure of $\mathbf{D}$ since:

$$\lim_{\tau_i^2\to+\infty}\left(\sigma^2\frac{(\mathbf{Z}^T\mathbf{Z})^-}{\tau_i^2} + \frac{\mathbf{D}}{\tau_i^2}\right) = \mathbf{C}_i, \quad i = 0, 1. \tag{S1}$$

This is due to the fact that the limit of all the off-diagonal entries is 0 ($\lim_{\tau_0^2\to+\infty}\rho\tau_1/\tau_0 = 0$ and $\lim_{\tau_1^2\to+\infty}\rho\tau_0/\tau_1 = 0$), and the diagonal structure is equal to those in Theorem 1.

S6.2 `Stan` code to specify the GIG distribution

Unfortunately, the GIG distribution is not implemented in the `Stan` library. However, if a model with correlated random effects needs to be fitted, the following statement before the model syntax can be included:

```
functions{ // GIG prior: only integer lambda
   real GIG_lpdf(real y, int lambda, real delta, real gamma){
      real log_p;
         log_p = - lambda * log(gamma / delta) - log(2.0)
            - log(modified_bessel_second_kind(lambda, delta * gamma))
            + (lambda - 1.0) * log(y) - 0.5 * (delta * delta / y + gamma * gamma * y);
      return(log_p);
      }
}
```

Then, for instance, if the variance component `tau2_0` is declared in the `parameters` block, then a GIG prior can be specified in the `model` block as follows:

```
tau2_0 ~ GIG(lambda, delta, gamma);
```

noting that only integer values for the parameter `lambda` can be fixed.

References

B. Carpenter, A. Gelman, M. D. Hoffman, D. Lee, B. Goodrich, M. Betancourt, M. A. Brubaker, J. Guo, P. Li, and A. Riddell. Stan: a probabilistic programming language. *Grantee Submission*, 76(1):1–32, 2017.

E. Fabrizi and C. Trivisano. Bayesian conditional mean estimation in log-normal linear regression models with finite quadratic expected loss. *Scandinavian Journal of Statistics*, 43(4):1064–1077, 2016.

A. Zellner. Bayesian and non-Bayesian analysis of the log-normal distribution and log-normal regression. *Journal of the American Statistical Association*, 66(334):327–330, 1971.
